# Illumina Sequencing Reveals Aberrant Expression of MicroRNAs and Their Variants in Whitefish (*Coregonus lavaretus*) Liver after Exposure to Microcystin-LR

**DOI:** 10.1371/journal.pone.0158899

**Published:** 2016-07-08

**Authors:** Paweł Brzuzan, Maciej Florczyk, Alicja Łakomiak, Maciej Woźny

**Affiliations:** Department of Environmental Biotechnology, Faculty of Environmental Sciences, University of Warmia and Mazury in Olsztyn, Olsztyn, Poland; Laboratoire de Biologie du Développement de Villefranche-sur-Mer, FRANCE

## Abstract

Molecular analyses show that challenging fish with microcystin-LR (MC-LR) causes perturbations of microRNA (miRNA) signaling. However, the significance and scope of these alterations is currently unknown. To address this issue, we studied *miRNA* gene expression in the liver of juvenile whitefish, *C*. *lavaretus*, during 28 days of exposure to a subacute dose of MC-LR (100 μg·kg^-1^ body mass). Using genomic resources of Atlantic salmon (AGKD03), the mature miRNA library of Atlantic salmon (miRBase-21) and bioinformatics tools (sRNAbench), we discovered and annotated a total of 377 distinct mature miRNAs belonging to 93 families of evolutionary conserved miRNAs, as well as 24 novel mature miRNA candidates that were mapped to 14 distinct *S*. *salar* miRNA precursors. miRNA-Seq transcriptome profiling of liver tissues revealed differential miRNA expression in control and treated fish at 14 days (73 miRNAs were modulated) and at 28 days (83 miRNAs) of the treatment, subsequently validated by qPCR for nine selected differentially expressed miRNAs. Additional qPCR study confirmed the miRNA-Seq data and revealed consistent, aberrant miRNAs expression profile in the later phase of MC-LR hepatotoxicity (7–28 d). Functional annotation analysis revealed that the aberrantly expressed miRNAs have target genes involved in cytoskeletal remodeling, cell metabolism, cell cycle regulation and apoptosis; dysregulation of these processes in liver cells leads to cirrhosis and hepatocellular carcinoma in humans. To enable deeper insight into the molecular responses of liver cells in fish exposed to MC-LR, we expanded the miRNAome analysis by inclusion of miRNA variants (isomiRs) profiles, and we showed that the isomiR profiles of liver specific MiR122, and a few other miRNAs, correlated with MC-LR treatment. Given the importance of isomiRs for disease biology in mammals, we believe that further research focused on the miRNA isoforms will bring us closer to better understanding the molecular mechanisms of MC-LR hepatotoxicity.

## Introduction

Microcystin-LR (MC-LR) is one of the most commonly occurring toxin produced by cyanobacteria of the genera *Microcystis*, *Anabaena*, *Planktothrix*, *Chroococcus*, that has received worldwide concern in recent decades [1]. MC-LR poses an environmental stress and health hazard on aquatic ecosystems when heavy blooms of cyanobacteria occur in lakes, since it causes toxic effects in mammals [2,3], birds [4] and aquatic animals [5,6]. There are also reports suggesting carcinogenic risk to people via drinking water and consuming animals harvested from the polluted water reservoirs [7–9].

The toxin is taken up via active transport mechanism (bile acids), as well as through the intestinal epithelium [10], leading to its accumulation mainly in the liver and thus exerting hepatotoxic effects [11,12]. The hepatotoxicity of MC-LR have been confirmed by the evidence that MC-LR inhibits protein phosphatases which leads to excessive phosphorylation of cell proteins and induces cellular proliferation, resulting in tumor-promoting activity in hepatocytes [13]. Moreover, MC-LR was found capable of triggering oxidative stress in liver cells which results in alterations in the cytoskeleton, leads to a loss of cell shape, cellular damage and finally to the liver injury [11,14]. Not surprisingly, these destructive processes have been contrasted by evidence that MC-LR triggers cell specific repair programs such as cell cycle arrest [13] or apoptosis both *in vivo* [15] and *in vitro* [16]. However, the mechanisms that regulate the balance between the destructive, protective, or repair processes during the MC-LR toxicity remain unclear. Currently, concerns are focused mainly on identification of particular hallmarks of the aberrant processes leading to MC-LR induced-liver toxicity and disease (e.g.[17,18]).

The role of microRNAs (miRNAs) in these cellular processes is still unclear. These molecules are members of the RNA interference system, and have attracted great interest in toxicology because of their role in cellular responses to xenobiotic exposure [19,20]. miRNAs are ubiquitously expressed short RNAs approximately 22 nucleotides (nt) in length and are particularly suitable for studying grids of molecular processes underlying pathological alterations of cells and tissues at the gene and protein levels [19]. miRNAs have been shown to regulate a large variety of molecular functions, and have been associated to a variety of diseases [21,22].

The biogenesis of miRNAs in vertebrates typically involves transcription of primary miRNA transcript (*pri-MiRNA*) by RNA polymerase II followed by cleavage by the DGCR8/Drosha complex into stem-loop structured miRNA precursor molecule (*pre-MiRNA*); in this paper, miRNA genes and their various products follow a naming convention proposed by Desvignes et al. [23]. The *pre-MiRNA* hairpin is subsequently transported from the nucleus to the cytoplasm by Exportin-5/Ran-GTP [24,25]. Then in the cytoplasm, the Dicer enzyme cleaves off the loop of the *pre-MiRNA*, leading to the formation of mature miRNA duplex. One strand (mature strand) of the duplex is preferentially incorporated into the RNA-induced silencing complex (RISC), whereas the opposite strand (passenger strand), is degraded. The mature miRNA strand, associated with the RISC, recognizes target sites (typically in the 3'-UTR) in the mRNA and mediates them for degradation, destabilization or translational repression, depending on the degree of complementarity to the target [26–28].

Multiple distinct mature miRNA species, termed isomiRs, can arise from the same hairpin arm, as revealed by recent advances in miRNA-Seq transcriptome profiling. These sequence variants typically differ from the mature miRNA sequences currently available in the public miRNA database, miRBase [29]. The sequences differ in that they have different lengths and different 5' and 3' ends, thereby increasing the diversity and complexity of the miRNAome. Some of the isomiRs have non-templated nucleotides at their 3' ends. While the biological relevance of isomiRs is not fully understood, their levels may distinguish diseased from normal tissue much better than the archetype miRNAs [30–32]. Nonetheless, with the exceptions of isolated efforts [33,34], currently available information concerning miRNA and isomiR expression profiles, and knowledge of their associations with fish diseases remain limited.

The versatile role of miRNAs has sparked the interest of our group in utilizing miRNAs as potential diagnostic [35,36] and prognostic [37,38] biomarkers of MC-LR toxicity and liver injury in fish. In our initial study, we showed that 5 out of 8 miRNAs, MiRlet7c, MiR16a, MiR21a, MiR34a and MiR122, were deregulated in the liver of whitefish (*Coregonus lavaretus*) after short-term (2 d) exposure to MC-LR [35]. In 2013, we began a research project to explore in detail the course of pathological changes in the liver of whitefish during long-term (28 days), continuous exposure to MC-LR. At the histological level, we recently reported that following weekly repeated injections with MC-LR (at a dose of 100 μg·kg^-1^ body mass), the histology of the fishes' liver and biomarker levels in their plasma were drastically changed in a time-dependent manner [39]. Because miRNAs regulate many pathways and orchestrate integrated responses in liver cells, we hypothesized that the exposure to MC-LR may also induce long-term alterations in global miRNA expression that may be linked to the adverse effects of the toxin. So far, no research has investigated the association of global miRNA expression changes with MC-LR-induced hepatotoxicity in the coregonid fish, which are particularly vulnerable to the toxin in the wild [40].

Therefore, the aim of this study was to investigate the long-term effects of MC-LR exposure on global miRNA expression profile in the liver of whitefish. To address this issue, juvenile whitefish individuals were repeatedly injected with MC-LR at a subacute dose of 100 μg·kg^-1^ body mass. After 14 and 28 days of the exposure, we extracted total RNA from the fish livers and assayed the samples using small RNA sequencing (miRNA-Seq). Then, using the data and bioinformatics tools, we obtained extensive lists of differentially expressed miRNAs, their variants and expression changes in treated whitefish compared to controls. Finally, we used qPCR to confirm the miRNA-Seq results, and to obtain time-course expression profiles of the most aberrant miRNAs. This study reveals the comprehensive miRNAome of fish hepatic tissue and provides background for further identification and classification of miRNA-dependent pathways leading us to better understanding the molecular mechanisms of MC-LR hepatotoxicity and potential carcinogenicity.

## Materials and Methods

### Fish maintenance, exposure, and collection of samples

The procedures related to fish maintenance and exposure were conducted in late autumn 2013 at the Department of Salmonid Research in Rutki (Inland Fisheries Institute in Olsztyn; Poland). The whitefish individuals (*Coregonus lavaretus*, L) were obtained from Rutki broodstock, which was originally developed by the Department from andronomous whitefish collected in 90's from the bay of Pomerania, Poland [41]. All fish were housed and handled in compliance with widely accepted guidelines of laboratory animal care. The experiment was approved by the Local Ethical Commission in Olsztyn (resolution No. 100/2011 of 23rd November 2011). The fish were acclimated for two weeks in 800 L flow tanks supplied with well (underground) water at the flow of 600 L·h^-1^. Throughout the experiment, all the fish were fed with reduced feeding procedure dependent on the water temperature, caloric content of the feed, and predicted fish mass. Water temperature in the tanks ranged between 8–9°C and oxygen level was above 90% of saturation. The fish were deprived of food 2 days prior to exposure (intraperitoneal injection) or collection of samples.

The dose of MC-LR (100 μg·kg^-1^ of body mass) and set of the treatment periods (1/3, 1, 2, 7, 14, and 28 days) were based on the previous studies on effects of pure microcystins or biomass of blue algae on fish metabolism [11,42], and our experience in studying molecular and physiological responses of whitefish to this toxin [35,43]. MC-LR (purity ≥ 95%; HPLC) was obtained from Enzo Life Sciences (Enzo Biochem, Inc.; USA) and dissolved in saline solution (0.8% NaCl) as a solvent vehicle. The prepared solution contained 10 μg of MC-LR per 200 μL of volume set for each intraperitoneal injection.

In the beginning of the experiment (0 d), the juvenile whitefish individuals were sorted by their mass (~100 g; age 1+) and divided into the following experimental groups that were then kept in separate tanks: i) control (n = 24), ii) sham-control (n = 24), iii) treated with MC-LR at a dose of 100 μg·kg^-1^ body mass (n = 48). Prior to exposure, the fish were anesthetized by immersion in etomidate solution [44], and received an intraperitoneal injection of the MC-LR solution or pure saline solution (Control). Fish from the sham-control group were only pricked with a needle but they did not receive any injection. In order to maintain continuous exposure, the injections with MC-LR were repeated every 7 days of the experiment (i.e. after 7, 14, and 21 days).

**Fig 1:**
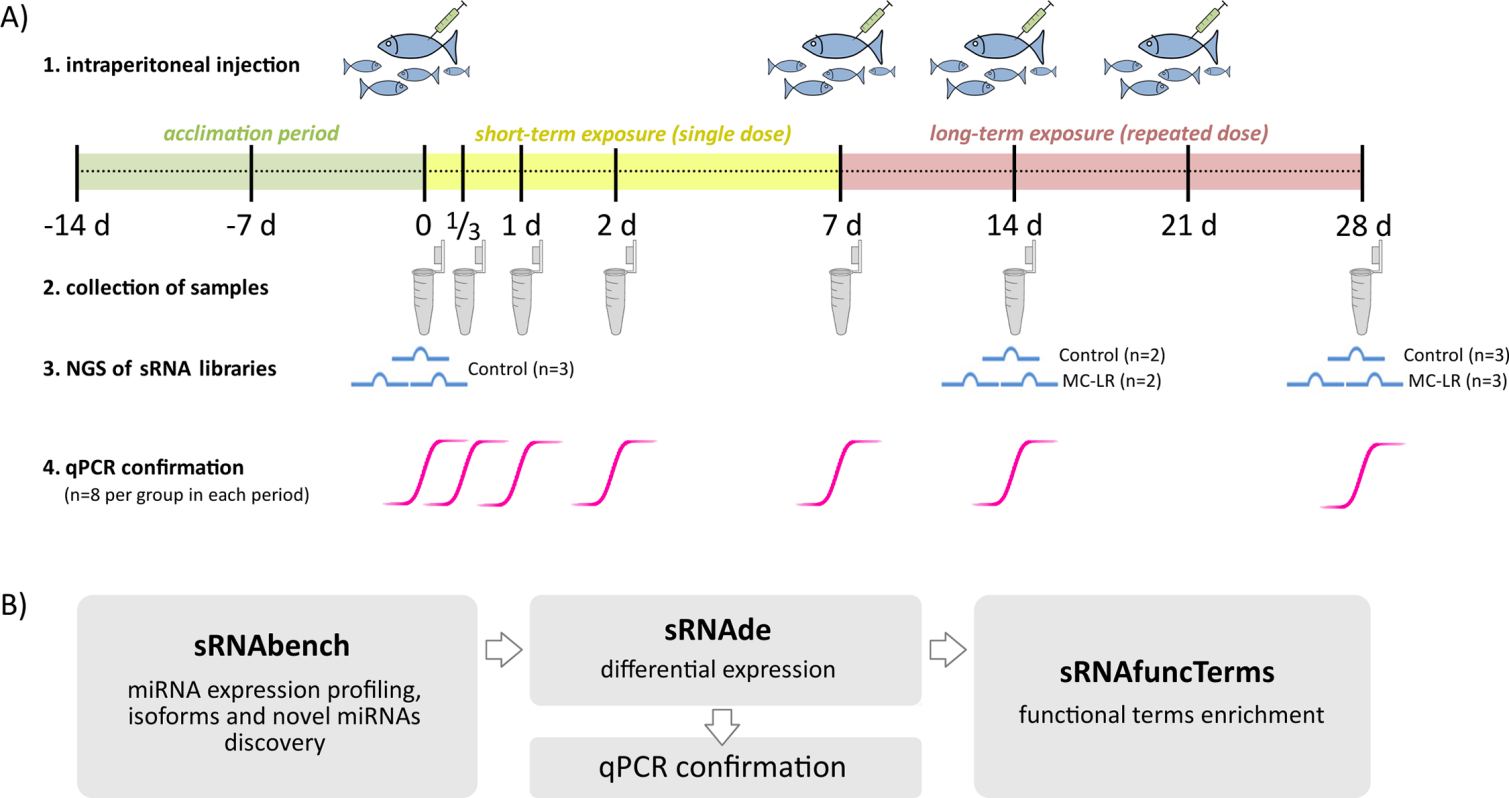
Experimental design for the discovery and characterization of miRNAs in whitefish challenged with microcystin-LR. Detailed explanations of the treatment experiment, sample collection times and bioinformatics tools used are provided in the text.

After each exposure period (1/3, 1, 2, 7, 14, and 28 days), randomly selected individuals (n = 4 from control or sham-control group, and n = 8 from the MC-LR-treated group) were anesthetized [45] and then euthanized by severing the spinal cord. After opening of body cavity, the liver was removed, then fragmented and immediately preserved in RNAlater according to the manufacturer's instructions (Sigma-Aldrich; Germany). The collected samples were stored at -20°C until used. Details of the study design were presented in the Fig 1.

### Total RNA extraction

Total RNA was extracted from the RNAlater-preserved liver (approximately 20 mg per sample) using a mirVana Isolation Kit (Ambion; USA) according to the manufacturer's protocol. Since we were particularly interested in studying the long-term effects of MC-LR exposure on global miRNA expression profile, we used total RNA samples extracted from the livers of control and exposed fish from the beginning of the experiment (0 d) and after 14 and 28 days of the treatment to prepare small RNA libraries and subsequently sequence them on Illumina platform.

### Library preparation and small RNA sequencing (miRNA-Seq)

The library construction and size separation were performed by Source BioScience (Nottingham, England) followed by sequencing analysis using Illumina HiSeq 2000 sequencing platform with 50 nt single reads (Illumina, USA). To ensure that the quantity and quality of the material submitted meet the specified criteria, the RNA concentration and purity were determined using a Bioanalyzer with the RNA 6000 Nano Assay (Agilent Technologies, Santa Clara, CA) following the manufacturer’s protocol. The Illumina® TruSeq Small RNA Sample Preparation Protocol (February 2014) was used to generate the small RNA libraries. This protocol works well for studying miRNA because it takes advantage of the structure of most small RNA molecules by ligating specific adapters to the 5`-phosphate and 3`-hydroxyl group, which are molecular signatures of their biogenesis pathway. The 3`and 5`adapters were ligated to the RNA and used as templates for reverse transcription to cDNA. To amplify the obtained cDNA, ligated samples underwent 13 cycles of PCR. For the size selection of amplified cDNA libraries, PCR products were then run on a 6% TBE gel with a custom ladder. The small RNA of approximately 140–160 bp in size was excised from the gel and incubated overnight. The incubated gel was eluted using a spin column. The obtained sequence libraries were then validated using an Agilent Bioanalyzer and the Qubit High Sensitivity Assay. One μl of the library was loaded onto the High Sensitivity chip to determine the size distribution of the library. Two μl of the library was used in the Qubit High Sensitivity Assay.

### Pre-processing of raw reads

The raw reads obtained from Source BioScience were processed to obtain clean reads by summarizing data production, evaluating sequencing quality or calculating the length distribution of small RNA reads. Sequence length distribution as well as the base calling accuracy as indicated by the phred quality score (Q score) were calculated with FastQC high throughput sequence data quality control software (Babraham Bioinformatics; UK; version 0.10.1). To assess overall miRNA-Seq data quality, reads with a Q score < 30 or without detectable adaptors were excluded from the data set. Adaptor sequences were trimmed from the 3`end using Cutadapt [46] with default adapter detection based on a 10% error rate. Reads without detectable adaptors were excluded from the data set. To avoid distortion and generation of false positive mappings by degraded RNA material and other small reads, the data set was depleted of all reads with a sequencing length of less than 16 nt and more than 36 nt. Reads were then sorted into a fasta formatted file and final read counts of unique sequences were generated by calling the sum of hits per miRNA by Tally, a component of the Kraken software suite [47].

### Profiling of miRNA sequences

To detect and profile miRNAs in our small RNA high-throughput sequencing data we used sRNAbench software [48]. The high-quality adaptor processed reads from 13 samples representing control and MC-LR exposed fish (Fig 1) were assigned in the genome mapping approach. In absence of genomic resources for *C*. *lavaretus*, in our miRNA analysis we based on data from genomic studies on Atlantic salmon, *Salmo salar*. Both fish species belong to Salmonidae family, whose common ancestor purportedly experienced a whole genome duplication event, and importantly they share 92% of expressed sequence tags contigs similarity [49]. The genome mapping mode first aligned all reads to the bowtie-indexed reference genome of Atlantic salmon (AGKD03; [50]), extracting the coordinates in internal BED format. Then, to profile the expression of miRNAs in whitefish samples, the short read aligner Bowtie was used to map sequence reads against the miRNA library (hairpin.fa + mature.fa) of Atlantic salmon [51] deposited in miRBase v. 21. A read was assigned to the reference RNA if its coordinates lay completely within the chromosome coordinates of the reference RNA [48]. Default commands were used in the sRNAbench analysis to allow for extensive profiling of all miRNA sequences and length variants (isomiRs) and prediction of novel miRNAs.

### Prediction of novel miRNAs

The characteristic hairpin structure of a miRNA precursor can be used to predict a novel miRNA. The method implemented in the sRNAbench tool is based on both structural and biogenesis features and has been used to predict novel miRNAs in rats [52]. The prediction software can detect those novel miRNAs for which both arms are represented by reads in the sample. Briefly, after clustering all reads, read clusters with distances of less than 60 nt are chosen. The *bona fide* miRNAs should have two read clusters corresponding to the two arms (two mature miRNAs) processed from the miRNA precursor. Then, the genomic sequence spanned by the two read clusters is extracted and its secondary structure and alignment pattern of the derived miRNA precursor is analyzed. Candidates are retained for which: i) the reads mapped to the stem of the putative miRNA precursor; ii) the reads do not fold back onto itself (i.e. it is not spanning the loop region); iii) all calculated values are below the thresholds set (see sRNAbench manual for details; http://bioinfo5.ugr.es/sRNAbench/sRNAbench_tutorial.pdf). Additionally, in order to detect those novel miRNAs for which only one arm can be detected the candidate generating and scoring method is used [48].

### IsomiR detection, classification and filtering

Most mature miRNAs do not only exist in their canonical form in the cell, but in a high number of different sequence variants, which are stably generated [53]. Sequence variants include 5’ and 3’ trimming and extension, non-templated additions (enzymatical addition of a nucleotide to the 3’ end, i.e. adenylation, uridylation). More recent analyses support the association of many of miRNA sequence viariants with the Argonaute silencing complexes and their functional roles through participation in the RNA interference pathway [30,32]. To detect isomiRs, sRNAbench: i) maps the reads to the genome or miRNA precursor sequences using the Bowtie seed option, ii) determines the coordinates of the mature miRNAs, iii) clusters all reads that map within a window of the canonical mature miRNA sequence, iv) applies a hierarchical classification schema [48]. Mapping was performed on the *S*. *salar* genome assembly AGKD03 [50], as described above. No insertions or deletions were allowed. To focus on the isomiRs of the present data set, we used the reference coordinates of the miRBase v. 21. We kept the isomiR molecules that were in the window of -6 to +6 nt of the reference mature sequences and had a total length of 16–28 nt, inclusive. To exclude lowly expressed isomiRs, we considered for statistical analysis, except for differential expression study, only isomiRs that had more than 100 reads in at least one sample. As the average number of total genome mapped reads for the samples in the study was 1.9 M reads, this corresponded to a threshold of about 52 transcripts per million. The filtering process yielded 987 isomiRs in the final expression matrix.

### Differential expression of miRNAs and isomiRs

For the detection of differentially abundant miRNAs and isomiRs in the samples we used the sRNAde module implemented in a sRNA toolbox ([54]; Fig 1). Two main results were generated. First, a heatmap for 20 most expressed miRNAs in the samples was calculated using hierarchical clustering which allowed to visualize the clustering of the 13 whitefish samples. Second, the differential expression was assessed using three frequently used programs: EdgeR, DESeq and NOISeq. To infer lists of differentially expressed miRNAs between control and MC-LR challenged whitefish at 14 d or 28 d of the treatment study using EdgeR method [55], the sRNAbench module generated expression matrices with TMM normalization [56,57]. False discovery rate (FDR) cutoff value for differentially expressed miRNAs between control and treated samples was set at FDR = 0.05. To yield lists of differentially expressed miRNAs with the other two methods, DeSeq and NoiSeq, the expression threshold was adjusted as p = 0.05, or set at the probability value of 0.8, respectively. To increase stringency of our approach consensus lists of differentially expressed miRNAs, detected by at least two methods, were determined. For the construction and visualization of the Venn diagrams with the numbers of differentially abundant miRNAs, Venn Diagrams software was used (http://bioinformatics.psb.ugent.be/software/details/Venn-Diagrams). Correlation patterns among isomiRs and samples were revealed using hierarchical clustering and Pearson method (Gene-E ver. 3.0.204; Broad Institute Inc.; USA). Statistically significant differences in the isomiR patterns were established with sRNAde module by defining first the isomiR ratio as the number of reads that belong to a given isomiR type divided by the total number of reads mapped to a given miRNA (canonical read count plus all isomiRs). Significant differences in the isomiR ratios between two conditions are then accessed by means of a standard t-test. In this case isomiR variants with FDR<0.05 were considered significantly different between control and treated samples.

### Confirmatory qPCR study and additional profiling of selected miRNAs

To confirm the miRNA-Seq data and to obtain more detailed profile of miRNA expression, we further used qPCR to examine the level of 9 selected miRNAs that were found to be the most aberrantly expressed in the liver of fish challenged to MC-LR. Briefly, total RNA samples were extracted from the livers of the control and the treated fish (after 1/3, 1, 2, 7, 14, and 28 days of the experiment) and further used in RNA polyadenylation and subsequent reverse transcription with the stem-loop primer [58] followed by Real-Time PCR. A detailed protocol of the used methods was given in the S1 File.

### Functional annotations of differentially expressed miRNAs

To gain insight into putative functions of the regulated miRNAs, we used sRNAfuncTerms tool (sRNA toolbox) with *Homo sapiens* as a reference species for miRNAs that were selected from those with statistically-significant differential-expression (Fig 1). In general, we determined the target genes and performed an enrichment/depletion analysis of functional annotations on the gene list, which gave insight into the molecular functions or biological processes of possibly regulated genes. sRNAfuncTerms uses Gene Ontology for functional annotations and is based on Annotation Modules to extract those functional annotations that are significantly enriched among the target genes [54].

## Results and Discussion

### Discovery and characterization of miRNAs in *Coregonus lavaretus*

The concentration and integrity of the total RNA extracted from livers of the 13 individuals (representing 3 control and 2 treatment groups) was sufficient to meet the quality requirements prior the miRNA-Seq (S1 Table). Following size separation and library preparation, the libraries were successfully subjected to small RNA sequencing using the Illumina HiSeq 2000 sequencing platform. The libraries were sequenced to a depth of 5.24·E^6^ to 1.11·E^7^ total trimmed reads (up to 36 nucleotides in length), yielding a total of 110 282 637 usable reads. The number of unique reads ranged from 91 468 to 523 015 per sample. The data from this study has been submitted to the NCBI SRA database (accession #SRP067625). The accession numbers for data from the individual samples, read numbers in the 13 samples, and concentrations of total RNA in the extracts are given in S1 Table.

**Fig 2:**
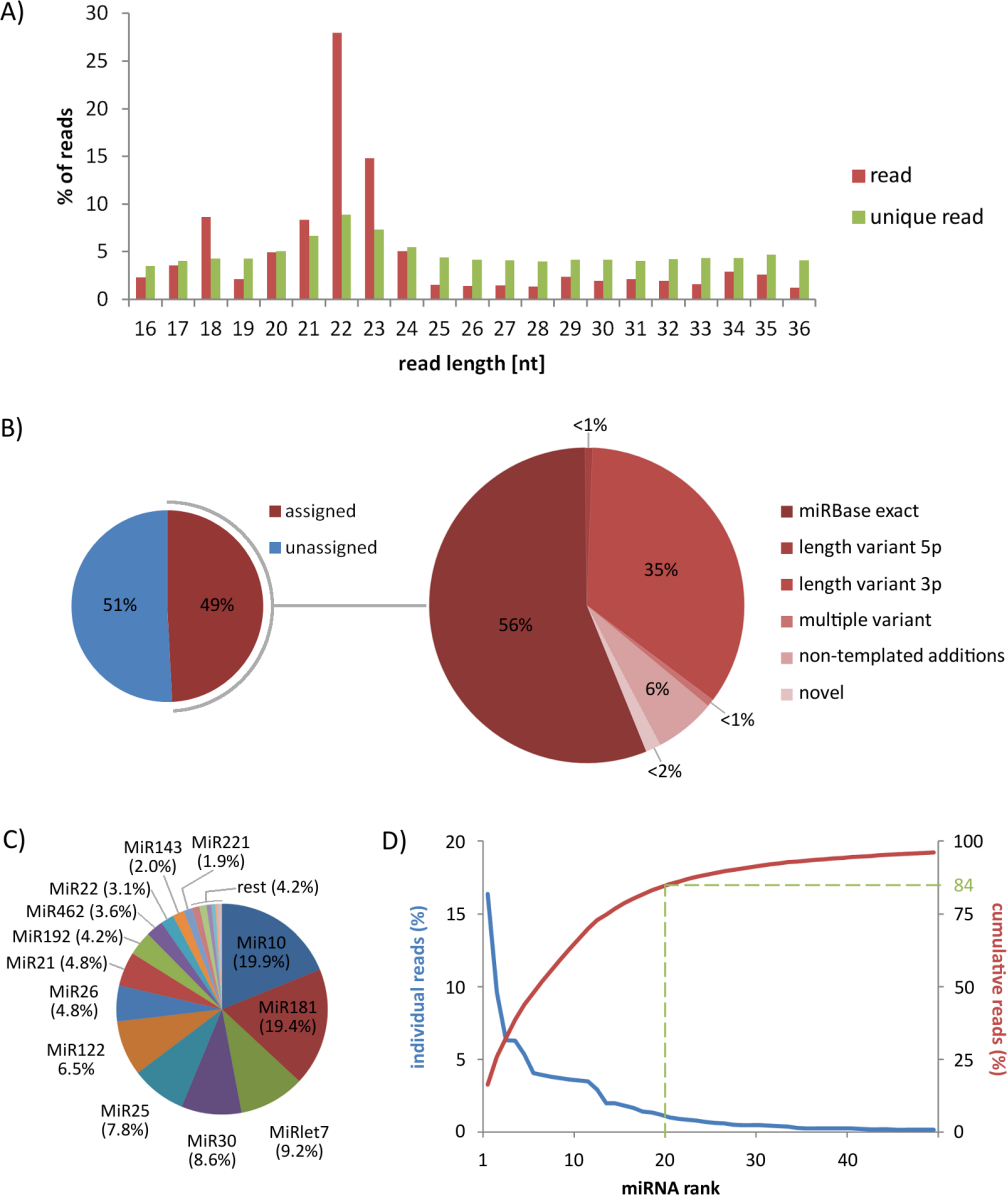
Identification of liver miRNAs from whitefish by Illumina sequencing. (A) Average length distribution for the reads derived from 13 liver samples of whitefish that were used for the miRNA expression profiling, isomiR and novel microRNA analysis. The largest mode of the reads is located at length 22. (B) Distribution of 25,007,856 mapped RNA sequence reads across miRBase v. 21 and the *S*. *salar* genome assembly AGKD03 (on left). The split of miRBase-exact, isomiRs and novel miRNA reads across miRNA annotations (on right). (C) Frequency of miRNA families as percentages of total number of miRNAs discovered. (D) Distribution of read counts across the fifty most abundant miRNAs. Contribution of individual miRNAs to total read count (blue), or cumulative read contribution (red) is plotted against ranked miRNA abundance. The dashed line shows the contribution of the 20 most abundant miRNAs in liver cells to all miRBase mapped reads.

All discovered miRNAs and their processing variants (isomiRs) were initially identified by sRNAbench analysis using a general mapping strategy and expression profiling [48]. The miRNA sequences from miRBase [59] were used to profile the expression of known miRNAs. To avoid non-miRNA derived reads in the output, both genome mapping and a library mapping mode were used. Briefly, the genome mapping mode first aligned all reads to the reference genome of the Atlantic salmon (AGKD03), extracting the coordinates in internal BED format. The coordinates were then compared to *S*. *salar* miRBase precursor sequences (371), or mature miRNA sequences (742)(miRBase v. 21). In general, a read was assigned to the reference RNA if its coordinates lay completely within the chromosome coordinates of the reference RNA. In summary, of a total of 38 854 902 input reads (100%) subjected to analysis, 25 007 856 reads (64.36%) mapped to the *S*. *salar* genome AGKD03 (S1 Table), and as expected, the mode of their read length was 22 nucleotides (nt)(Fig 2A). Of the ~25 M reads assigned to AGKD03, 12 302 565 (49.20%) reads mapped to miRBase-listed miRNA hairpins of *S*. *salar* (Fig 2B left). Of these, 12 112 217 reads (98.45% of total) were classified as exact (canonical, archetype) miRBase sequences or isoforms (isomiRs; Fig 2B right). Apart from exact (canonical) miRNA sequences, which constituted 55.96% of total miRBase-listed salmon miRNAs, the analysis revealed a number of isomiRs with 5’ or 3’ trimming or extensions (36.20%) and non-templated additions (6.27%)(Fig 2B right). The additions are added after Dicer processing through miRNA maturation [60], and may affect miRNA stability and target selection, thus contributing to interactions between miRNA and mRNA [53]. The rest of the genome assigned sequence variants 190 348 reads (1.55%) belonged to novel miRNA candidates (Fig 2B right).

A total of 377 distinct evolutionary conserved mature miRNA sequences (184 5p-miRNA and 193 3p-miRNA sequences) were identified in whitefish liver in the two-step approach, together with 239 putative precursor sequences (*pre-miRNAs*) at positions within the assembly of the *S*. *salar* genome (AGKD03). An overview of all evolutionary conserved mature 5p and 3p, precursors (*pre-miRNA*), and families of the miRNAs identified is given in S2 Table. The annotation of miRNAs showed that we identified 93 evolutionary conserved miRNA families in the liver of whitefish. The most abundant miRNA families were MiR10 (19.91%), MiR181 (19.36%), MiRlet7 (9.24%), MiR30 (8.61%), MiR25 (7.81%), and MiR122 (6.54%), followed by MiR26 (4.78%), MiR21 (4.76%) and MiR192 (4.21%) (Fig 2C). The 20 most abundant miRNAs in liver cells of whitefish contributed 84% of all miRBase-mapped reads (Fig 2D). The expression levels of miRNAs in whitefish varied greatly, with some produced at only a few copies per sample (or absent), and others present in thousands of copies. In all the samples, the median read number of the 377 detected miRNAs was 217, and in individual samples, the median ranged from 43 to 109. This high variability in miRNA copy numbers in tissues is similar to that in previous reports of animal miRNAomes, including fishes, the Atlantic halibut [33], Atlantic salmon [51] and rainbow trout [61,62]. Furthermore, the type of evolutionary conserved *miRNA* genes present in a species is expected to be in accordance with its taxonomic level [63]. According to the current miRBase repository, all 93 miRNA families identified in the current study were reported for teleost fish species. Unfortunately, more in-depth comprehensive evolutionary analysis of the current data is not possible due to the lack of a whitefish genome resources.

One of the advantage of conducting bioinformatics analysis with sRNAbench software is that it allows for discovery of novel miRNAs. Such data may improve our knowledge of the miRNA diversity in tissues and their role in coordinating molecular networks in cells. In the current study, a total of 24 new mature miRNA candidates were mapped to 14 distinct precursor miRNA sequences that were encoded in the genome of *S*. *salar* but were not yet registered for *S*. *salar* in the miRBase (Table 1; S3 Table). These were annotated in accordance with the nomenclature rules of Kozomara and Griffiths-Jones [59], using the name, family number and suffixes of the miRNA in other species, for which the novel miRNA candidate does have putative homolog (hairpin = true and homolog = true)(S3 Table). Interestingly, further screening of the mature miRNA sequences against recently published miRNA database for rainbow trout [61,62] revealed that all but one pre-miRNA from Table 2, *pre-mir34*, are present in the rainbow trout miRNAome. Thus, ongoing studies are identifying more and more miRNAs in salmonid fish species which show a great degree of structure and functional conservation. Not surprisingly, the newly discovered mature miRNAs in the current study belong to miRNA families that play regulatory roles in pathways of signal transduction, apoptosis and cell cycle. For example, MiR34a, a member of the MiR34 family, has been one of the most extensively studied miRNAs since the observation that its up-regulation causes growth arrest, apoptosis, and senescence [64,65]. Further studies have confirmed its role as a tumor suppressor in various types of cancers [66,67]. In contrast, some other miRNAs listed in the Table 1 have been reported as oncogenes, e.g. MiR187 [68], MiR193 [69], or MiR454 [70].

**Table 1:** Novel mature miRNA candidates identified in this study.

miRNA 5p	mature 5p (*C*. *lavaretus*)	miRNA 3p	mature 3p (*C*. *lavaretus*)	*pre-miRNA*	*pre-miRNA* sequence (*S*. *salar*)^1^	Putative biological process
MiRlet7g-5p	ugagguaguuguuuguacagu	MiRlet7g-3p	cuguacaagccacugccuugcu	*pre-miRlet7g-1*	5'-ugguc**ugagguaguuguuuguacagu**uugagggucugugauucugcccaauacaggagcuaacuguacaagccacugccuugccuaggg	signal transduction
MiRlet7g-5p	ugagguaguuguuuguacagu	MiRlet7g-3p	cuguacaagccacugccuugcu	*pre-miR*let7g-2	5'-ugguc**ugagguaguuguuuguacagu**uugaggguccgugauucugcccgauaaaggagcuaacuguacaagccacugccuugccuaggg	signal transduction
MiR457a-5p	uagcagcacaucauuacuggua	MiR457a-3p	ccaguauggcuugugcugcucca	*pre-miR457a*	5'-ggcgu**uagcagcacaucauuacuggua**gcugccccuucucugggcugccaguauggcuugugcugcuccagucag	cell proliferation
MiR222b-5p	acccaguacucaguguaaggucug	MiR222b-3p	agcuacaucugauuacuggguca	*pre-miR222b*	5'-ggcucacccaguacucaguguaaggucugugugaucaaaguac**agcuacaucugauuacuggguca**guaau	cell proliferation, cell differentiation
MiR223-5p	aguguauuugacaagcugaguuggacacu	MiR223-3p	ugucaguuugucaaauaccccaa	*pre-miR223a*	5'-cacuu**aguguauuugacaagcugaguuggacacu**caguaugacagggugucaguuugucaaauaccccaaguga	hematopoiesis
MiR223-5p	guguauuugacaagcugaguuggacacu	MiR223-3p	ugucaguuugucaaauacccca	*pre-miR223b*	5'-cacuuaguguauuugacaagcugaguuggacacucaguaugacaggg**ugucaguuugucaaauacccca**aguga	hematopoiesis
MiR34a-5p	uggcagugucuuagcugguugu	MiR34a-3p	aaucagcaaguauacuaccgca	*pre-miR34*	5'-uucuc**uggcagugucuuagcugguugu**ugugagggagagaacgaagcaaucagcaaguauacugccgcagaaac	cell cycle, signal transduction
MiR193-5p	ugggucuuugcgggcaagguga	MiR193-3p	aacuggccuacaaagucccagu	*pre-miR193*	5'-gagucugggucuuugcgggcaaggugaguccucauucauuc**aacuggccuacaaagucccagu**uucug	cell proliferation
MiR30d-5p	uguaaacauccccgacuggaaucu	MiR30d-3p	cuuucagucagguguuugcugc	*pre-miR30d-1*	5'-ggggc**uguaaacauccccgacuggaaucu**guucucuaucaucagagcuuucagucagguguuugcugcugcug	cell differentiation
MiR30d-5p	uguaaacauccccgacuggaaucu	MiR30d-3p	cuuucagucagguguuugcugc	*pre-miR30d-2*	5'-ggggc**uguaaacauccccgacuggaaucu**guucuacaucaucagagcuuucagucagguguuugcugcugcug	cell differentiation
MiR187-5p	ggcugcaacacaggacauggg	MiR187-3p	ucgugucuuguguugcagccagu	*pre-miR187-1*	5'-ccaggggcugcaacacaggacaugggagcuaccugccccuccugc**ucgugucuuguguugcagccagu**ggaac	cell proliferation
MiR187-5p	ggcugcaacacaggacaugggagc	MiR187-3p	ucgugucuuguguugcagccag	*pre-miR187-2*	5'-ccagg**ggcugcaacacaggacaugggagc**uaccuguccuuccugc**ucgugucuuguguugcagccag**uggaac	cell proliferation
MiR338a-5p	aacaauauccuggugcuguaugagu	MiR338a-3p	uccagcaucagugauuuuguuac	*pre-miR338*	5'-cuggg**aacaauauccuggugcuguaugagu**gaguggggaaaagcuccagcaucagugauuuuguuacuagg	cell differentiation
MiR454-5p	acccuaucaguauugucucugc	MiR454-3p	uagugcaauguugcuuauaggguu	*pre-miR454*	5'-cugaa**acccuaucaguauugucucugc**uguccacuuugaguagugcaauguugcuuuuaggguuucagu	signal transduction, cell proliferation

^1^The short processed miRNA with largest number of read counts is indicated by bold letters within precursor sequences. The loop part of the sequence is underlined.

This table is based on predictions from mapping the reads obtained from *Coregonus lavaretus* samples to the stems of the putative *pre-miRNA* sequences of the *Salmo salar* genome (AGKD03; whole genome shotgun sequencing project). The table shows novel mature 5p and and/or 3p sequences along with corresponding precursor (*pre-miRNA*) sequences and implicated putative biological processes for a given miRNA.

**Table 2:** MicroRNAs with statistically significant isomiR ratio differences (FDR< 0.05) between control and challenged fish after 28 days of the MC-LR treatment.

isomiR pattern(n)	miRNA	Mean	SD	Mean	SD	P	FDR
**Length variant 3p(148)**	MiRlet7b-5p	0.428	7.44E-06	0.281	2.14E-04	1.49E-04	0.022
	MiR30d-5p	0.599	8.55E-05	0.427	3.47E-04	3.06E-04	0.023
	MiR30c-5p	0.338	3.40E-04	0.149	2.25E-04	3.59E-04	0.018
	MiR30b-5p	0.754	3.06E-04	0.577	3.40E-04	5.87E-04	0.022
	MiR25-3p	0.253	5.01E-05	0.171	1.52E-04	1.20E-03	0.036
	MiR27b-3p	0.631	4.38E-05	0.524	3.17E-04	1.32E-03	0.032
	MiR122-2-3p	0.414	1.23E-03	0.230	7.17E-05	1.97E-03	0.042
	MiRlet7i-5p	0.283	9.74E-05	0.219	5.82E-05	1.97E-03	0.036
	MiR122-5p	0.241	1.24E-04	0.093	1.04E-03	3.63E-03	0.048
**Non-templated A addition(123)**	MiR125b-5p	0.021	2.52E-07	0.015	2.01E-07	2.54E-04	0.031
**Non-templated AA Addition(49)**	MiR122-5p	0.001	5.45E-09	0.000	9.65E-09	3.48E-04	0.017
**Non-templated C Addition(86)**	MiR30d-5p	0.047	3.52E-06	0.032	1.10E-07	3.57E-04	0.031
**Non-templated UUUU Addition(6)**	MiRlet7b-5p	0.002	7.60E-08	0.000	1.80E-07	6.26E-03	0.038

n, the number of miRNAs in the analysis; SD, standard deviation; p, the exact p-value (t-test); FDR, the FDR corrected p-value.

### MC-LR exposure altered miRNA expression profiles

Although a number of individual molecular pathways in liver cells have been implicated or confirmed in animal responses to MC-LR, there has been no investigation of global changes in the miRNA profile of the liver of MC-challenged fish. To address this issue, we conducted a 28 day study with whitefish and collected liver samples of control and challenged fish for miRNA profiling and differential expression analysis. Throughout the experiment, there were no mortalities in the MC-LR-treated individuals or the control fish.

**Fig 3:**
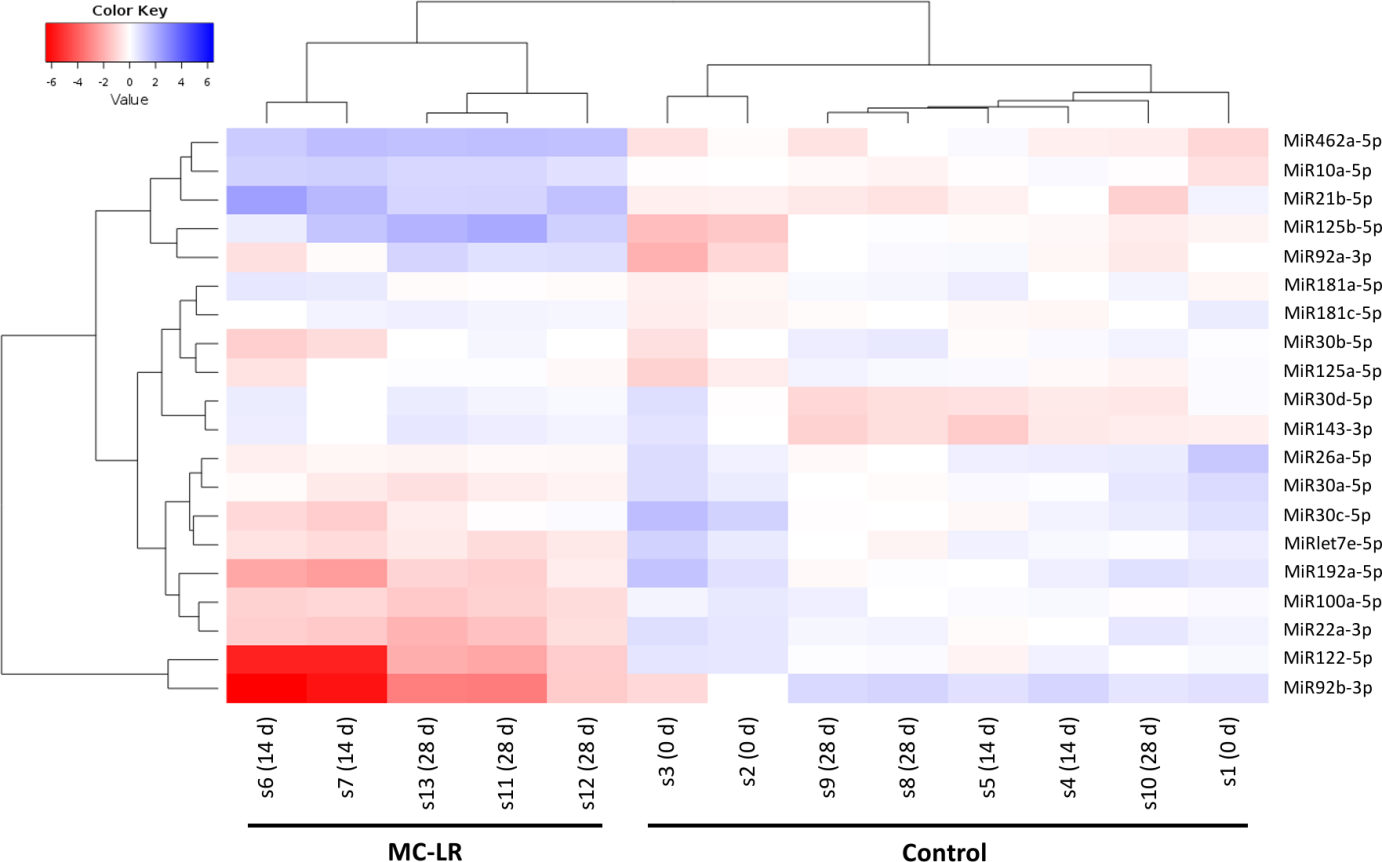
Heatmap for 20 top expressed miRNAs. The map was generated using log_2_ of median normalized read counts per million. Dendrograms on the left and above the heatmap show correlations (Pearson) from hierarchical clustering analysis on the miRNAs and samples, respectively.

Differential expression analysis of the 13 liver samples from the control and the treated whitefish revealed that MC-LR significantly altered miRNA expression profiles. Not surprisingly, hierarchical clustering analysis with the 20 top expressed miRNAs showed that their expression profile in the MC-LR group differed from that in the control group (Fig 3). Of the top expressed miRNAs, 5 were up-regulated and 15 were down-regulated. The up-regulated miRNAs were MiR21b-5p, MiR10a-5p, MiR125b-5p, MiR462a-5p, and MiR92a-3p. The down-regulated miRNAs included MiR92b-3p and MiR122-5p, which both showed at least 4-fold decrease (Fig 3).

**Fig 4:**
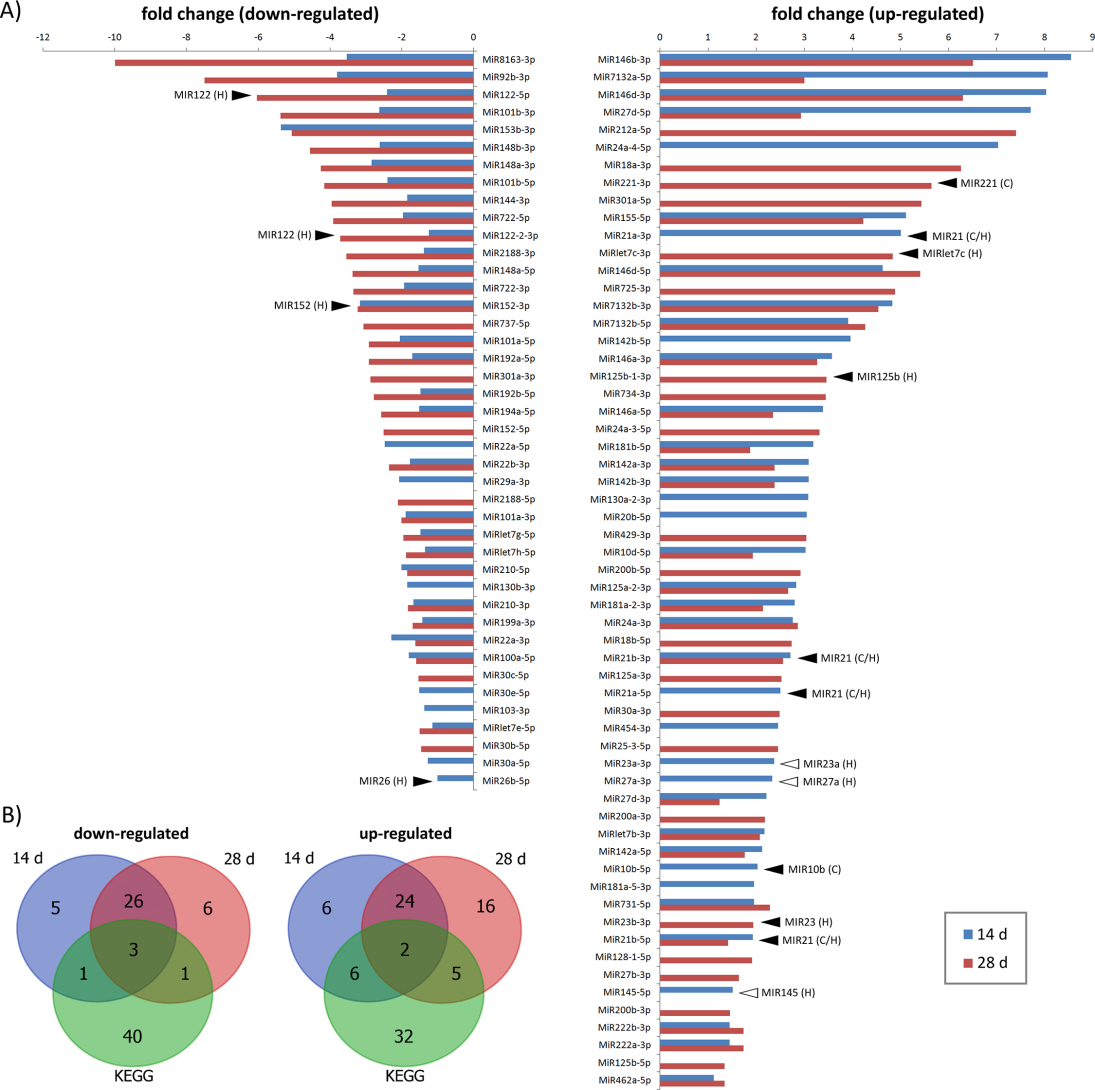
Differentially expressed miRNAs in the liver of whitefish on 14 d and 28 d of the treatment. (A) Fold changes (log_2_) of miRNAs found deregulated by at least two methods used in the current study for differential expression analysis of RNA-seq data. Bars present mean expression (n = 2 for 14 d; n = 3 for 28 d) relative to control group of the respective time point (14 or 28 d). Arrowheads denote whitefish miRNAs for which human counterparts are involved in processes (KEGG data) leading to cirrhosis and hepatocellular carcinoma, indicated as (C) and (H), respectively. Solid arrowheads denote whitefish and human miRNAs sharing the same direction of the expression, whereas empty arrowheads indicate the opposite expression changes. (B) The Venn diagrams illustrating the relationship between sets of differentially down- or up-regulated miRNAs in the study and those deposited in KEGG database.

RNA sequencing showed that the studied experimental groups (Control 0 d, Control 14 d, Control 28 d, MC-LR 14 d, and MC-LR 28 d) differed slightly in their mature miRNA composition. Of the 377 mature, evolutionary conserved miRNAs discovered, 205 were found in all groups. On the other hand, there were several miRNAs that were only found in certain groups of samples (S1 Fig). In all, 223 mature miRNAs were detected for differential expression analysis using sRNAde software [54]. S2 Fig shows numbers of differentially expressed miRNAs obtained with three different methods: EdgeR, DeSeq and NoiSeq. The three methods yielded somewhat different results with respect to both the numbers and the lists of differentially expressed miRNAs (S2 Fig; S4 Table), reflecting their individual performances and biases with regard to the data [71]. Therefore, in further analysis we used only those miRNAs that were detected as differentially expressed by at least two methods. As for the control groups, miRNA expression profiles were very similar between samples, except for Control 0 d and Control 28 d, for which 3 differentially expressed miRNAs were found (S2 Fig). In contrast, strong differences between differentially expressed mature miRNAs in the treated group and their respective controls were noticed (Fig 4A). After 14 d of the treatment, a total of 73 miRNAs were differentially expressed, of which 38 were up-regulated (10 were time-point specific; tps) and 35 down-regulated (6 tps). After 28 days of the treatment, 83 miRNAs were modulated, of which 47 were up-regulated (21 tps) and 36 down-regulated (7 tps). Several up-regulated (MiR21a-3p, MiR125b-1-3p, MiR23a-3p, MiR10b-5p, MiR221-3p) and down-regulated (MiR92b-3p, MiR152-5p, MiR122-2-3p or MiR122-5p) miRNAs shown in Fig 4A have been implicated in cirrhosis and hepatocellular carcinoma in humans [31]. To find out how many of the miRNAs deregulated in these whitefish (Fig 4A) are also differentially expressed in diseased human liver, we compared our data with publicly available KEGG information reporting miRNAs involved in hepatitis, cirrhosis and hepatocellular carcinoma (http://www.genome.jp/kegg-bin/show_pathway?hsa05206). The two sets of data shared 5 significantly down-regulated and 13 significantly up-regulated miRNAs (Fig 4B). These findings provide evidence that subsets of *miRNA* genes, including *mir10*, *mir122*, and *mir92*, are commonly deregulated in vertebrate liver tissue and can potentially underlie initiation and progression of destructive processes in liver cells.

### Potential role of differentially expressed miRNAs in MC-LR–induced pathological liver changes

**Fig 5:**
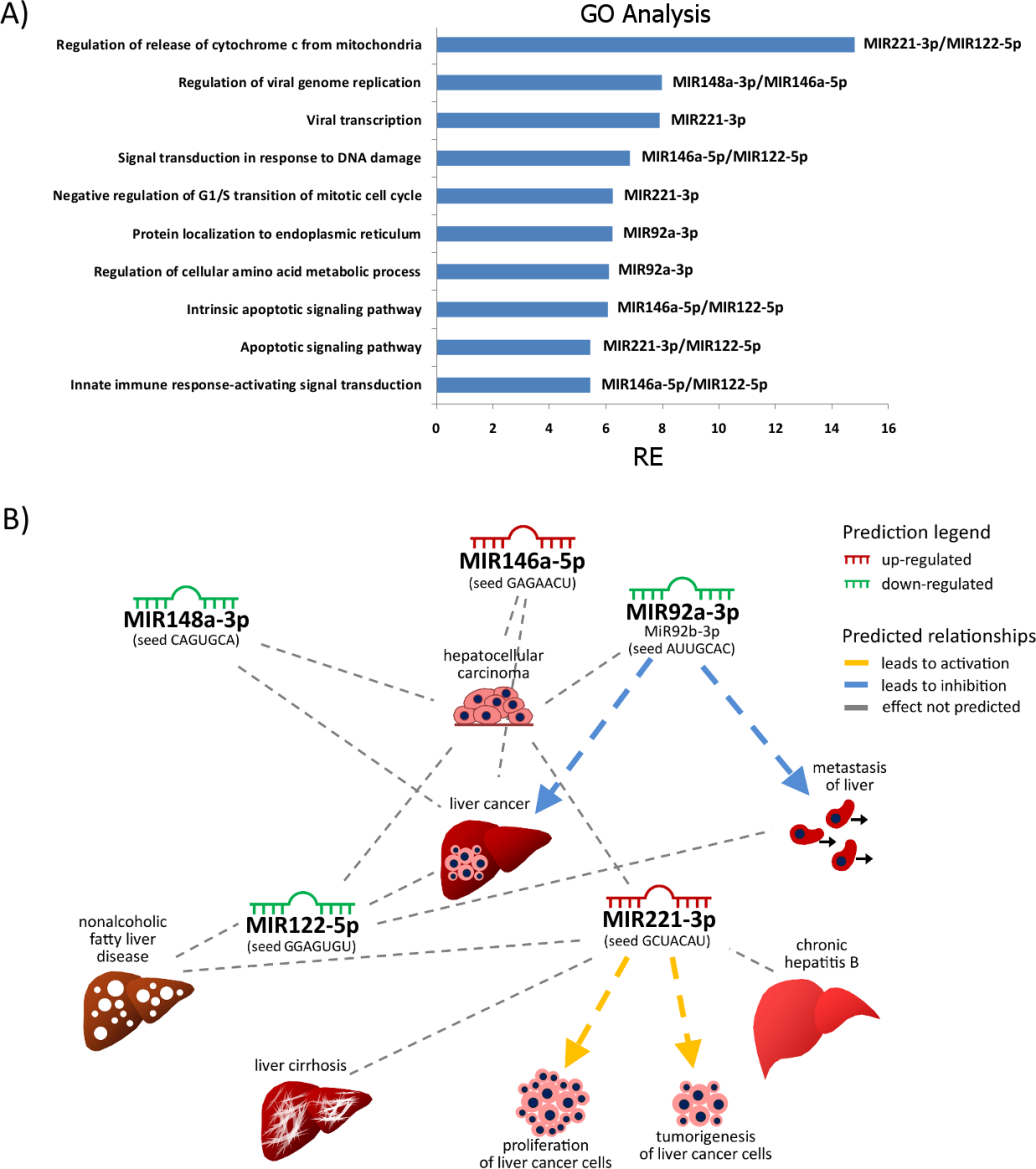
Potential pathologic contribution of miRNAs to diseased state of liver in whitefish exposed to MC-LR. (A) Top ten functional GO terms (Biological process) of the 5 *miRNA* genes found deregulated in diseased liver of both human and whitefish (MC-LR). RE; Relative enrichment with respective miRNA modules [54]. (B) A simplified network showing the relationship among 5 miRNAs (MIR92a(b)-3p, MIR122-5p, MIR146a-5p, MIR148a-3p, MIR221-3p) found to be deregulated in the diseased human liver (Ingenuity® Pathway Analysis) and in the liver of whitefish exposed to MC-LR (this study).

To analyze the biological significance of the differentially abundant miRNAs, potentially involved in regulatory pathways, we performed an enrichment/depletion analysis of functional annotations on the list of putative target genes using sRNAfuncTerms module of the sRNA toolkit [54]. We focused on 6 aberrantly expressed miRNAs found in our study (MiR122, MiR92a(b), MiR146a, MiR148a, MiR221) for which contributions to a disease state of liver in humans have been proven. The analysis revealed that the aberrantly expressed miRNAs have target genes involved in cytoskeleton remodeling, cell metabolism, cell cycle regulation and apoptosis, among the top 10 enriched pathways (Fig 5A). MIR146a in particular has gained importance as the modulator of differentiation and function of cells of the innate and adaptive immune system [72]. On the other hand, down-regulated MIR148a triggers processes of cell proliferation, progression and migration in hepatocelluar carcinoma [73,74]. Moreover, we have recently reported that in whitefish, MC-LR repeatedly injected at a dose 100 μg·kg^-1^ cause severe liver injury at early period, which results in marked loss of the liver function (i.e. low plasma protein, icterus, fibrosis) after 14 and 28 days of exposure [39]. With regards to the importance of the MIR146a and MIR148a in regulating key cellular processes in the diseased liver state (Fig 5B), we believe that the modulation of the miRNA expression in the liver of whitefish after 14 and 28 days of the exposure may be linked to the chronic effects of MC-LR exposure [39] and the potential activity of the cyanotoxin as a tumor promoter [1].

**Fig 6:**
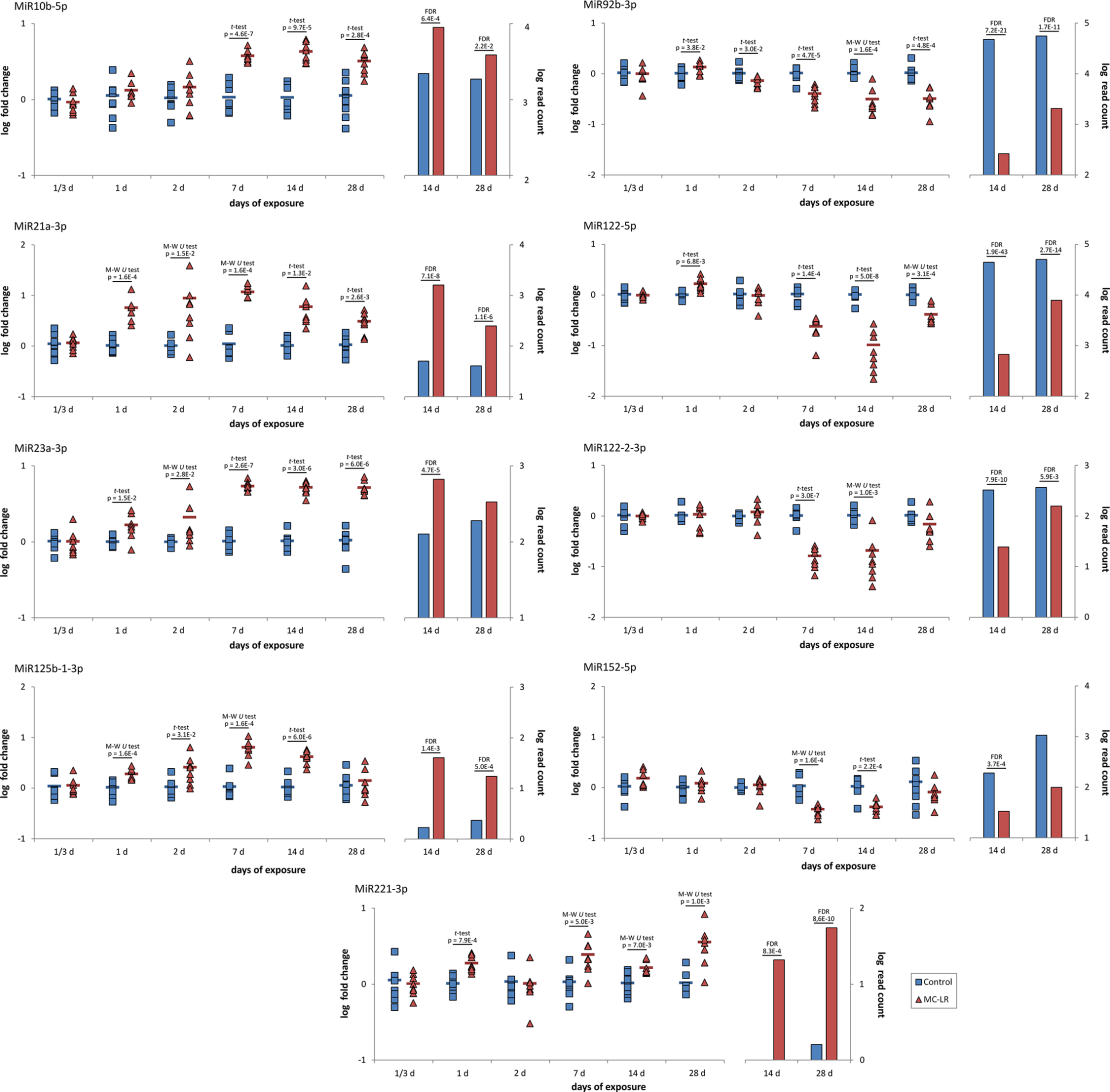
Microcystin-LR induces aberrant expression of microRNAs in the liver of whitefish. Changes in 9 selected miRNAs expression (log fold change) assessed using qPCR (left panel) and miRNA-Seq method (right panel) in control (blue squares) and microcystin-LR-treated (100 μg MC-LR kg^-1^ body weight; red triangles) whitefish. Point markings in the left panel present values for fish individuals within a group normalized by MiR181a-5p and MiRlet7b-5p as reference miRNAs, whereas horizontal lines indicate mean values (n = 8) of expression, relatively to control group of the respective time point (1/3 through 28 d). Comparisons between groups were performed using independent *t*-test and significant p-values are shown (IBM SPSS Statistics v.23). In addition, Mann-Whitney *U* test was used for pairwise comparisons of groups with non-normally distributed data (Kolmogorov-Smirnov test; p < 0.05). Bars in the right panel indicate median values of read counts for the miRNAs in control and MC-LR treated group after 14 d (n = 2) and 28 d (n = 3) of the treatment. FDR values from comparisons of TMM normalized data from individuals representing respective groups are shown above the bars.

To further confirm the miRNA-Seq data and to explore how differentially abundant miRNAs were expressed throughout the whole treatment study, we designed a qPCR study that covered the 14–28 days period and additionally extended the analysis to earlier sampling points of the treatment (1/3–7 d). Towards this end, we examined the expression of 9 differentially expressed miRNAs which have been previously linked with dysregulation of signal pathways in diseased liver of vertebrates. Except MiR122-2-3p, MiR23a-3p, and MiR125b-1-3p after 28 days, qPCR analysis (Fig 6; left panel) of the samples from the 14th and the 28th day of the treatment showed high concordance with miRNA-Seq (Fig 6; right panel). The small discrepancies between qPCR and miRNA-Seq for the three miRNAs could probably arise due to a relatively small number of samples examined by Illumina platform and a poor FDR control of the EdgeR method with regard to the samples/conditions [71]. In terms of the miRNA expression level, the control group and the MC-LR–treated group did not differ significantly after 1/3 d of the experiment (Fig 6). At 1 d, there was significantly more of the miRNAs in the MC-LR group than in the control group, with the exception of MiR10b-5p, MiR122-2-3p, and MiR152-5p. From day 2 of the exposure period to day 28, all but one of the examined miRNAs followed two distinct trends: one group of miRNAs was up-regulated (MiR10b-5p, MiR21a-3p, MiR23a-3p, MiR125b-1-3p, MiR221-3p), while the other group was down-regulated (MiR92b-3p, MiR122-5p, MiR122-2-3p, MiR152-5p). These differences were mostly significant, especially at 7 and 14 d. The presence of differentially abundant miRNAs in whitefish liver in the later phase of MC-LR treatment (especially at 14d, 28 d) suggest a role for them in the molecular mechanism of MC-LR chronic toxicity (Fig 5B).

Particularly, MiR122 is one of the most important liver-specific miRNAs [75], which in our study was consistently downregulated after exposure to MC-LR (Fig 6). In mammals, MIR122 is involved in maintaining an adult-liver phenotype by suppressing the expression of non-liver genes [76], and it is well recognized as a tumor suppressor, since loss of its function leads to hepatocarcinogenesis [77]. It has been suggested that downregulation of MIR122 expression may be involved in mediating intrahepatic bile duct injury or liver fibrosis [75]. In fish, studies suggest its role with silencing genes involved in cholesterol degradation and excretion [34]. In contrast to MiR122 expression profile, MiR21 and MiR221 were upregulated after exposure to MC-LR (Fig 6). In the literature, both specimens are well recognized as oncogenic miRNAs and their expression was found to be induced in different types of cancer cell, including hepatocellular carcinoma (e.g. [78,79]). Our understanding of the miRNAs' functions in the molecular mechanisms of the fishes' liver health and disease is far from complete, and only few papers exist possibly linking MC-LR-induced hepatotoxicity and an abnormal profile of global miRNA expression. However, MIR122, MIR21, and MIR221, collectively, have been reported to be aberrantly expressed after MC-LR exposure in the mice and human liver cells, both *in vivo* and *in vitro* [80,81]. Interestingly, the consistent expression pattern of the miRNAs (MiR122↓, MiR21↑, MiR221↑) found in our study is in compliance with the previous reports. If the liver metabolic networks are evolutionarily conserved between species and connected by specific miRNAs (such as MiR21, MiR122, or MiR221), our results suggest severe consequences for the liver cell maintenance and may provide molecular background explaining how MC-LR affects biological systems on a larger scale. Nonetheless, neither mRNAs directly targeted and repressed by the miRNAs in fish, nor the detailed phenotype of the altered gene regulation in the liver of whitefish exposed to MC-LR, are currently known to lengthen the discussion in this manner.

### IsomiR characterization and expression profiles

An individual *miRNA* gene may give rise to miRNAs of different length, named isomiRs, that been shown to be functionally relevant [29]. In the current study, we extended the miRNAome analysis to the isomiR profiles in both control and MC-LR treated whitefish. After we catalogued all the isomiRs that were abundant in our material, we focused on the following questions: (i) Are the whitefish isomiRs similar to mammalian ones with regard to their 5' and 3' end variation? (ii) Do the isomiR profiles allow for discrimination between the control and the treated samples? (iii) Do isomiR abundances correlate with their length?

### IsomiRs exhibit more variation in their 3´ ends

**Fig 7:**
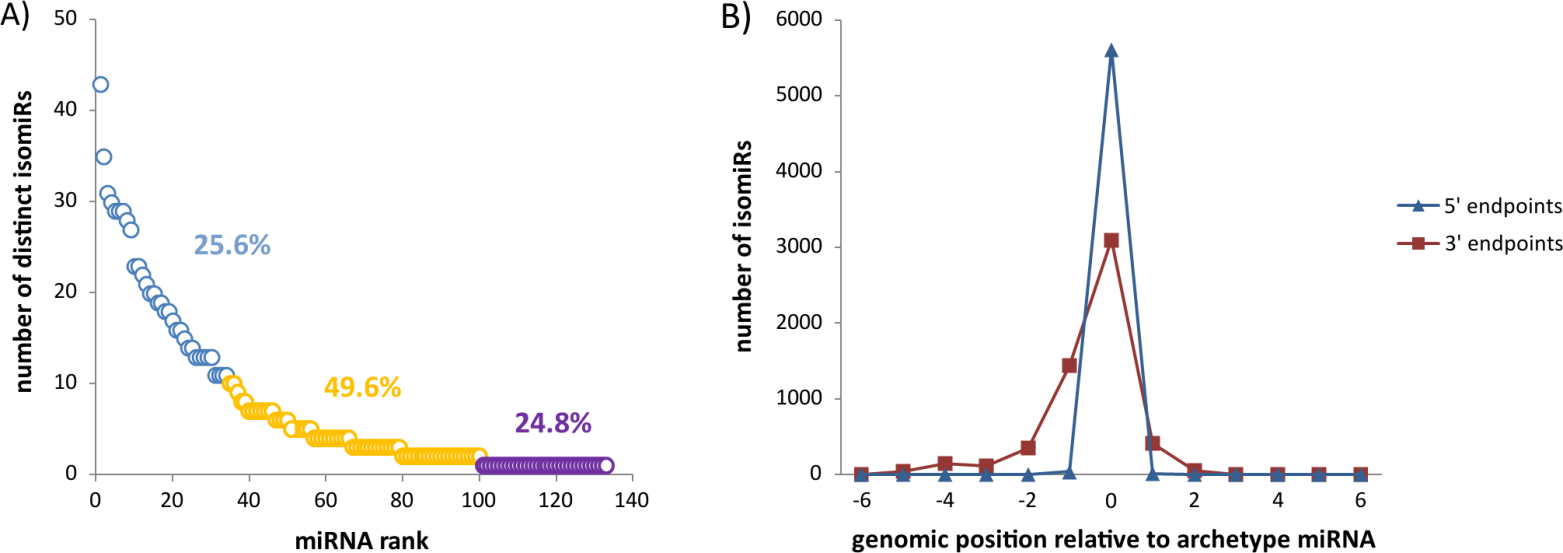
General characteristics of the isomiRs. (A) The number of distinct isomiRs per microRNA. (B) Distribution of the number of isomiR endpoints at each genomic position relative to the archetype’s coordinates.

Fig 2B shows frequency of major isomiR variants in the 13 studied liver samples of whitefish before filtering procedure. Looking at the proportions of the isomiR reads assigned to different isomiR classes in individuals samples (S3 Fig), we found that the most abundant were 3p length variants (82% of all isomiR reads), followed by A-non-templated additions (7%) and U-non-templated additions (about 4%). The remaining 7% of all isomiRs detected included 5p length variants and other nucleotide variant isomiRs. After isomiR filtering, which left us with only isomiRs that had more than 100 reads in at least one sample, we found a total of 133 hairpin arms producing 987 distinct isomiRs (the full data set is included in S5 Table). Of the 987 unique isomiRs that we identified, 369 (37%) show templated additions/deletions at the 3´-end, whereas only 29 (3%) show templated additions/deletions at the 5´-end. Non-templated additions were detected in 259 distinct isomiRs (26%), 129 isomiRs (13%) carried nucleotide variants, whereas 78 (8%) showed multiple sequence variants of the canonical miRNAs (123; 13%) detected in the study. On average, there are 7 (7.42) distinct isomiRs that arise from each hairpin arm. However, this number exhibits significant variation as can be seen from Fig 7A: 33 (24.8%) hairpin arms produce a single miRNA molecule, half the arms (66 of 133, or 49.6%) produce between 2 and 10 isomiRs, and 34 arms (25.6%) produce more than 10 isomiRs. Among the latter, MiR122-5p had the most isomiRs (43 isomiRs) followed by MiR26a-5p (35 isomiRs) and MiRlet7a-5p (31 isomiRs). It is noteworthy that after the filtering procedure, we found 10 arms that did not produce the exact, archetype miRNA and that 3 of them produced only one other (non-archetype) isomiR. Finally, we plotted the distribution of the 5p and 3p termini of the isomiRs around the termini of the archetype miRNA (Fig 7B). Generally, the 5' ends show a narrower range (+1/-2 nt) of modifications, compared to 3' endpoints (+3/-6 nt). Levene’s test indicated that the variances in the two distributions differ in a statistically significant manner (p = 0.022) with the 3p termini of the isomiRs exhibiting more diversity than the 5p termini. This is probably due to functional pressure on the 5' end resulting in higher end-region conservation. The above observations confirm earlier findings in different species and tissues [30,82] and indicate that reference molecules (i.e. canonical miRNAs) capture only a portion of the diverse repertoire of miRNA products that can arise from a given precursor arm.

### Liver specific MiR122-5p and MiR122-2-3p isomiR profiles correlate with MC-LR treatment

**Fig 8:**
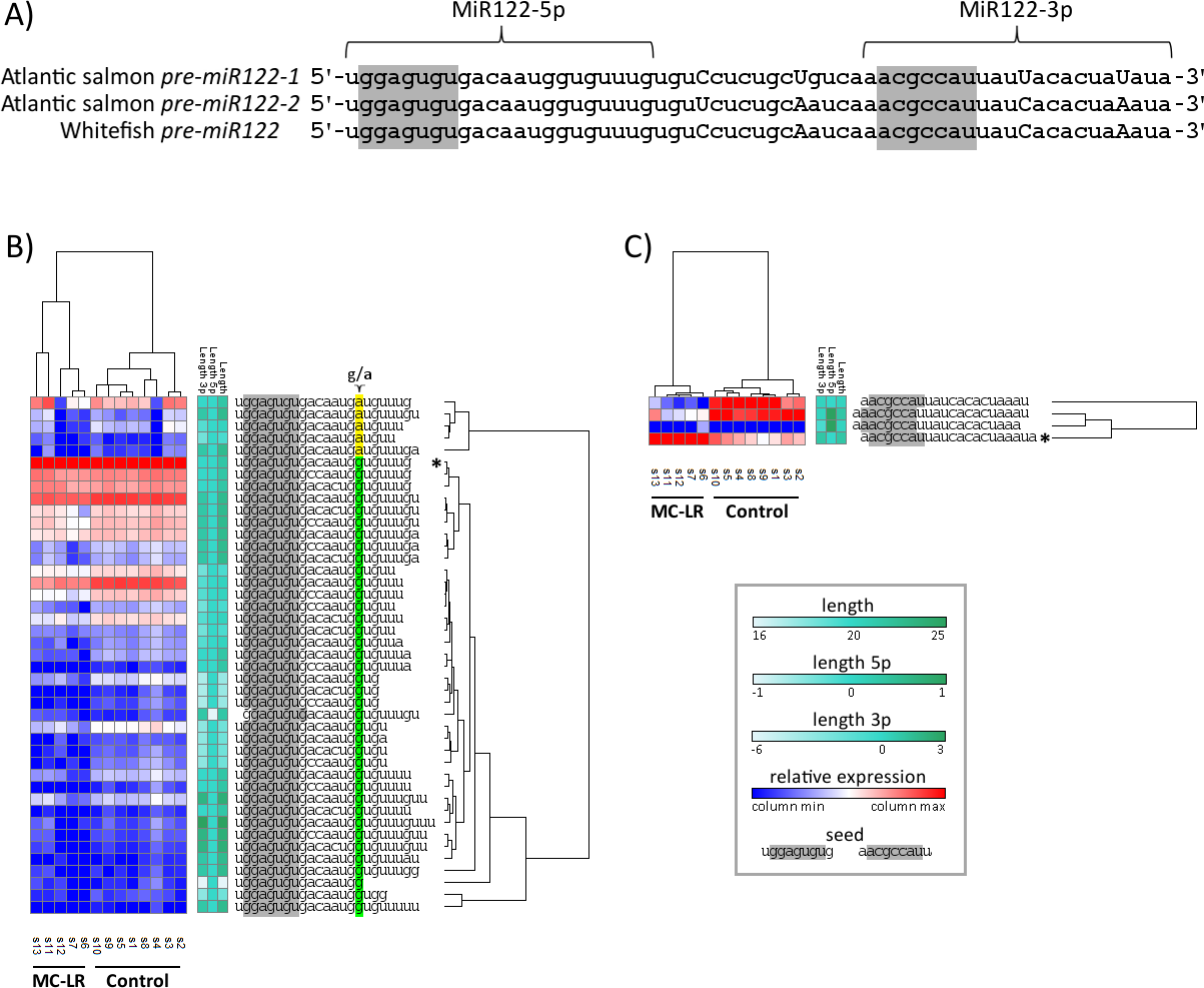
Sequence differences and correlation patterns between multiple MiR122 isomiRs detected in whitefish. (A) Nucleotide differences, marked with capital letters, within *pre-miR122* sequences from whitefish and Atlantic salmon. The data suggest that the isomiRs detected in the current study are products of two *mir122* loci, *mir122-1* and *mir122-2*. (B) (C) Hierarchical clustering (HCL) analyses (1-Pearson correlation) on the isomiRs 122-5p and 122-2-3p, respectively. Each row represents one isomiR between Control and MC-LR treated samples. Colors in the heatmaps represent normalized read count number of each individual isomiR. The separation of the MC-LR treated from Control samples is evident in the two cases. MiR122-5p isomiRs are clustered based on specific characteristics including the relative 3' endpoints and the length, and G/A difference in the mature sequence. These are discussed in the main text. Asterisks mark the archetype miRNAs.

When examining isomiR profiles in the control and MC-LR treated samples at the end of experiment (28 d), we found that some miRNAs exhibited treatment-dependent isomiR ratios (Table 2). With respect to 3p length variants, of a total 148 distinct miRNAs filtered for the analysis, 9 miRNAs differed significantly between groups (FDR < 0.05), and another four differed with respect to non-templated A additions (MiR125b-5p and MiR122-5p), non-templated C additions (MiR30d-5p), and non-templated U additions (MiRlet7b-5p). In all cases, the treatment of fish with MC-LR resulted in a decreased number of the reads for respective isomiR variants (Table 2). In further analysis, we were particularly interested whether expression profiles of miRNA variants of liver specific MiR122 can be distinguished between the groups of MC-LR challenged and healthy fish (S6 Table); we selected these miRNA loci because they produce a considerable number of isomiRs that we can use to interpret the correlation patterns. To this end, we clustered the 13 samples analyzed in the study, based on the Pearson correlation between respective numbers of isomiRs. Fig 8 shows that 43 isomiR variants of the MiR122-5p (Fig 8B) and four isomiRs of the MiR122-2-3p (Fig 8C) distinguish control from the MC-LR treated samples.

Even though the correlation patterns of either MiR122-5p or MiR122-2-3p isomiRs argue for the importance of the findings, the biological implications of the isomiR categories and pathways regulated is not currently understood. However, it is reasonable to think that the process of isomiR generation in whitefish may not be random, implying some regulatory mechanisms that may influence the spectrum of isomiRs in the cells. First, since salmonids (salmon and whitefish) have undergone an additional whole genome duplication compared to many other animal species [49], the isomiR pattern may be a result of different expression bias of *mir122* gene paralogues; two *S*. *salar* sequences putatively expressed from two *mir122* loci are presented in Fig 8A. In general, *miRNA* loci differ considerably in the level of expression, of either 5p or 3p isomiRs, or, alternatively the profiles are derived from the arm switching [29]. Since the isomiRs' endpoints and length may be determinants of the regulation of their expression, we examined whether these specific characteristics of isomiRs (i.e. the relative 3' endpoint and the length) determines correlation pattern of the isomiRs. Thus, we focused on the MiR122 isomiR profiles from the 5p arm which exhibited an aberrant expression profile in the studied liver samples. As seen in Fig 8B, the hierarchical analysis revealed that the isomiR cluster comprises two subgroups: one subgroup contained 38 isomiRs, which shared the same 15 and 16 nt (-GA-) as the canonical miRNA and formed clusters based mainly on the isomiR 3' length; the second group contained isomiRs of different 3' lengths, but sharing double G at nucleotide position 15 and 16 of the archetype MiR122-5p. It is unlikely that the–GA- isomiR sequences carry the G-to-A mismatches due to sequencing or alignment errors [83], bearing in mind that more than 100 sequencing reads were analyzed and 13 cycles of PCR where used when preparing the libraries. Similarly dubious seems that the -GA- isomiRs have resulted from co-expression of two allelic variants from one heterozygous *mir122* locus. Most likely, the G/A difference which occurs in the region outside the seed of the mature MiR122-5p sequence suggests rather the–GA- isomiRs represent expression products from two homozygous *mir122* genetic loci carrying either nucleotide. Likely, the miRNA sequence heterogeneity and end region length diversity may have functional meaning and further studies should resolve causes of the observed isomiR repertoire in whitefish liver.

Although isomiRs are rarely studied small RNA regulatory molecules, there are reports showing their comprehensive biological roles (e.g. [84]). A particular hallmark of miRNAs is their association with the Ago silencing complex that coordinates downstream gene-silencing events. Although multiple mature products are expressed from a given miRNA precursor arm, it is not clear how many of them are actually loaded onto the silencing complex. To investigate this, cross-linking immunoprecipitation (CLIP) and photoactivatable ribonucleoside-enhanced cross-linking and immunoprecipitation (PAR-CLIP) of the Ago protein is accomplished to identify the miRNA-mediated recruitment of different transcripts to RISC (the major component of the RNA-induced silencing complex). Target mRNAs of miRNAs co-precipitate with Ago and can thus be identified using different platforms [85]. If findings of the current study show that miRBase reference entries and the newly identified isomiRs are represented at roughly equal levels in the Ago-Par Clip dataset for whitefish (not available at the moment), this will suggest that in addition to the miRBase reference miRNAs, the isomiR variants can also be functionally active. Such a study would yield also information whether different isomiRs from the same precursor arm can work cooperatively to repress target genes or whether they have different targeting profiles that result in increased diversity of the targeted transcripts [30,84]. There is the possibility that they may underlie various aspects of liver injury and regeneration (e.g. differences in the onset triggers and progression of the liver pathological changes).

## Conclusions

Compared to environmental toxicant-associated epigenetic changes found in studies on animal models [86], the relationship between environmental toxicant exposure, miRNA alterations, and disease risk in fish is understudied. The current study reveals the comprehensive miRNAome of whitefish hepatic tissue and provides biological knowledge for investigation of miRNA-dependent pathways in fish, following the treatment with an environmental toxin, microcystin-LR. The MC-LR exposure causes perturbations on several hepatic miRNA signaling pathways, and the expression profiles of miRNAs and their variants may become suitable markers in tracking adverse effects in liver cell. As more miRNA profiling studies with other toxins and other organisms are performed, it will be possible to obtain a connectivity grid highlighting miRNAs that drive hepatotoxic effects. This will greatly facilitate future studies on vertebrate miRNAs in the context of liver diseases.

## Supporting Information

S1 Fig The number of *miRNA* genes expressed in the liver of whitefish. The diagram demonstrates *miRNA* genes that are uniquely expressed in each group, with the overlapping regions showing the number of miRNAs that are expressed in two or more groups.
(DOCX)

S2 Fig Analysis of differentially expressed miRNAs. Numbers of differentially expressed miRNAs (n = 223 miRNAs) detected by EdgeR, DeSeq and NoiSeq methods. (A) The table, above the diagonal, for each pairwise comparison the three values shown give the numbers of differentially expressed miRNAs obtained with EdgeR, DeSeq and NoiSeq, respectively. NoiSEq expression threshold was set at the probability value of 0.8, while those of EdgeR and DeSeq there were adjusted p-values <0.05. Below the diagonal the consensus result, i.e. the number of differentially expressed miRNAs detected by the three methods. (B) The Venn diagrams illustrating the relationships between the sets of differentially expressed miRNAs detected by the three methods after 14d and 28 d of the treatment. Yellow bates indicate comparison of samples from the MC-LR–treated group with the respective control group.
(DOCX)

S3 Fig Multiple sequence variants and non-templated additions of all isomiRs detected. Proportions of 3’,5’, multiple sequence variants and non-templated additions (G, C, T, A) of all isomiRs detected in samples s1 through s13.
(DOCX)

S1 File Confirmatory qPCR study. (DOCX)

S1 Table Summary of samples sequenced for discovery of *Coregonus lavaretus* miRNAs. (DOCX)

S2 Table Complete data of miRNA expression in the liver of whitefish. Summary at the read level of miRBase mapped (exact) miRNAs across 13 liver samples (s_1 through s_13) of whitefish examined in the study. The table contains columns with individual, precursor and family miRNA names for *Salmo salar* (miRBase_21), the read counts (RC) and the reads per million (RPM), the reads normalized by the total number of genome mapped reads.
(XLSX)

S3 Table Novel mature miRNA candidates in *Coregonus lavaretus* identified in the study. This table is based on predictions from mapping the reads of *Coregonus lavaretus* samples to the *Salmo salar* genome sequence (AGKD03; *Salmo salar*, whole genome shotgun sequencing project; Andreassen et al. 2013). Shown are precursor (*pre-miRNA*) sequences identified, and their structures, along with corresponding novel mature 5p and and/or 3p sequences. 1 Bona-fide microRNA: both arms have been found showing a perfect Drosha/Dicer pattern (2mt 3' overhang allowing for +/- 1nt); Homologous: Non bona-fide, but a putative homologous microRNA exists (the sequence mapped to a known sequence from miRBase ver. 21).
(XLSX)

S4 Table Differential expression data obtained with different methods. Full results of the analysis (Full_table_) with EdgeR, DESeq and NOISeq method in control and MC-LR treated group after 14 d (n = 2) and 28 d (n = 3) of the treatment. To yield significantly differentially expressed miRNAs (Selection_), for the EdgeR method false discovery rate was set at FDR = 0.05, for the DESeq the expression threshold was adjusted at p = 0.05 and for the NOISeq the probability value was set at 0.08.
(XLSX)

S5 Table The number of distinct isomiRs per microRNA. (XLSX)

S6 Table The set of filtered miRNA variants of liver specific MiR122. Only isomiRs that had more than 100 reads in at least one sample were considered.
(XLSX)
