# Supplementary material for: Illumina Sequencing Reveals Aberrant Expression of MicroRNAs and Their Variants in Whitefish (Coregonus lavaretus) Liver after Exposure to Microcystin-LR
Source: PLoS One. 2016 Jul 8;11(7):e0158899. doi: 10.1371/journal.pone.0158899 (PMC4938405; doi:10.1371/journal.pone.0158899)
Supplement: S1 Fig — The diagram demonstrates miRNA genes that are uniquely expressed in each group, with the overlapping regions showing the number of miRNAs that are expressed in two or more groups. (DOCX) [file pone.0158899.s001.docx]

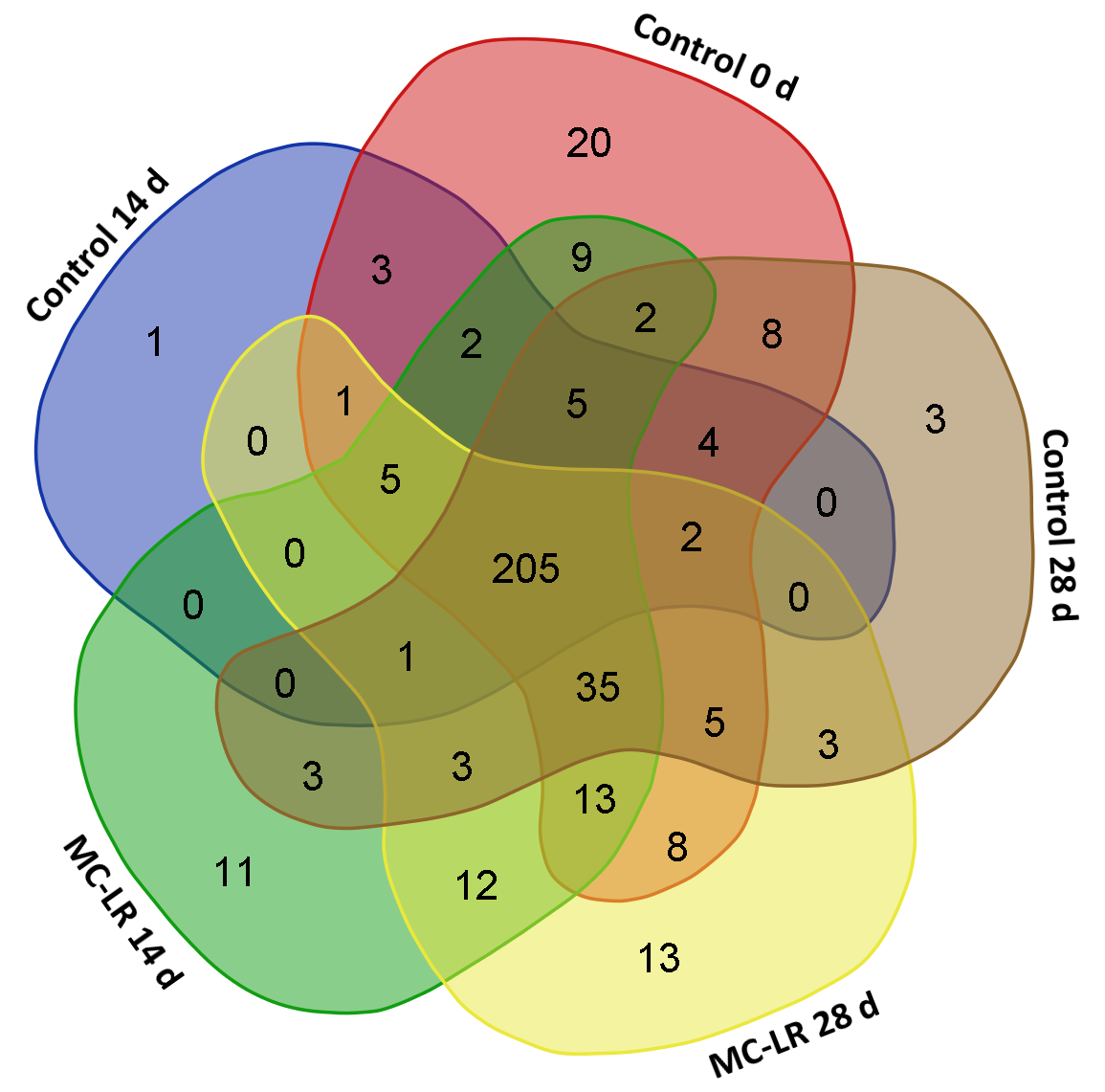


**S1 Fig.** **The number of *miRNA* genes expressed in the liver of whitefish.** The diagram demonstrates *miRNA* genes that are uniquely expressed in each group, with the overlapping regions showing the number of miRNAs that are expressed in two or more groups.
