# Supplementary material for: Illumina Sequencing Reveals Aberrant Expression of MicroRNAs and Their Variants in Whitefish (Coregonus lavaretus) Liver after Exposure to Microcystin-LR
Source: PLoS One. 2016 Jul 8;11(7):e0158899. doi: 10.1371/journal.pone.0158899 (PMC4938405; doi:10.1371/journal.pone.0158899)
Supplement: S2 Fig — Numbers of differentially expressed miRNAs (n = 223 miRNAs) detected by EdgeR, DeSeq and NoiSeq methods. (A) The table, above the diagonal, for each pairwise comparison the three values shown give the numbers of differentially expressed miRNAs obtained with EdgeR, DeSeq and NoiSeq, respectively. NoiSEq expression threshold was set at the probability value of 0.8, while those of EdgeR and DeSeq there were adjusted p-values <0.05. Below the diagonal the consensus result, i.e. the number of differentially expressed miRNAs detected by the three methods. (B) The Venn diagrams illustrating the relationships between the sets of differentially expressed miRNAs detected by the three methods after 14d and 28 d of the treatment. Yellow bates indicate comparison of samples from the MC-LR–treated group with the respective control group. (DOCX) [file pone.0158899.s002.docx]

| Samples | Control  (0 d) | Control  (14 d) | Control  (28 d) | MC-LR  (14 d) | MC-LR  (28 d) |
| --- | --- | --- | --- | --- | --- |
| Control  (0 d) | –– | 0  0  0 | 17  0  4 | 138  94  38 | 131  79  26 |
| Control  (14 d) | 0 | –– | 0  0  0 | **103**  **70**  **32** | 111  50  11 |
| Control  (28 d) | 0 | 0 | –– | 126  100  38 | **114**  **84**  **19** |
| MC-LR  (14 d) | 38 | **29** | 37 | –– | 38  5  5 |
| MC-LR  (28 d) | 25 | 10 | **18** | 2 | –– |

B)

A)


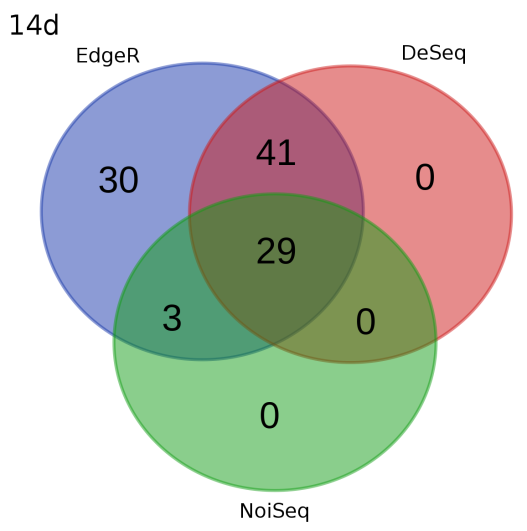

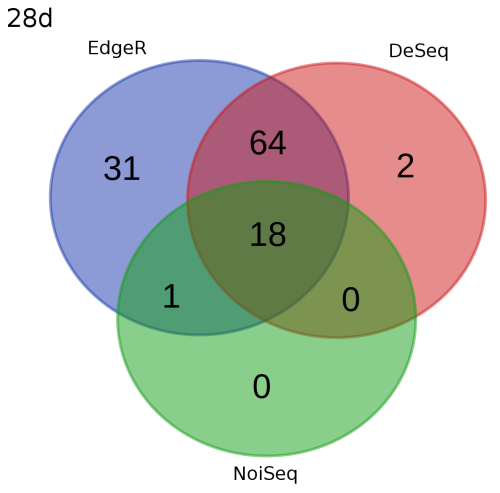


**S2 Fig.** **Analysis of differentially expressed miRNAs.** Numbers of differentially expressed miRNAs (n=223 miRNAs) detected by EdgeR, DeSeq and NoiSeq methods. (A) The table, above the diagonal, for each pairwise comparison the three values shown give the numbers of differentially expressed miRNAs obtained with EdgeR, DeSeq and NoiSeq, respectively. NoiSEq expression threshold was set at the probability value of 0.8, while those of EdgeR and DeSeq there were adjusted p-values <0.05. Below the diagonal the consensus result, i.e. the number of differentially expressed miRNAs detected by the three methods. (B) The Venn diagrams illustrating the relationships between the sets of differentially expressed miRNAs detected by the three methods after 14d and 28 d of the treatment. Yellow bates indicate comparison of samples from the MC-LR–treated group with the respective control group.
