# Supplementary material for: Illumina Sequencing Reveals Aberrant Expression of MicroRNAs and Their Variants in Whitefish (Coregonus lavaretus) Liver after Exposure to Microcystin-LR
Source: PLoS One. 2016 Jul 8;11(7):e0158899. doi: 10.1371/journal.pone.0158899 (PMC4938405; doi:10.1371/journal.pone.0158899)
Supplement: S3 Fig — Proportions of 3’,5’, multiple sequence variants and non-templated additions (G, C, T, A) of all isomiRs detected in samples s1 through s13. (DOCX) [file pone.0158899.s003.docx]

**
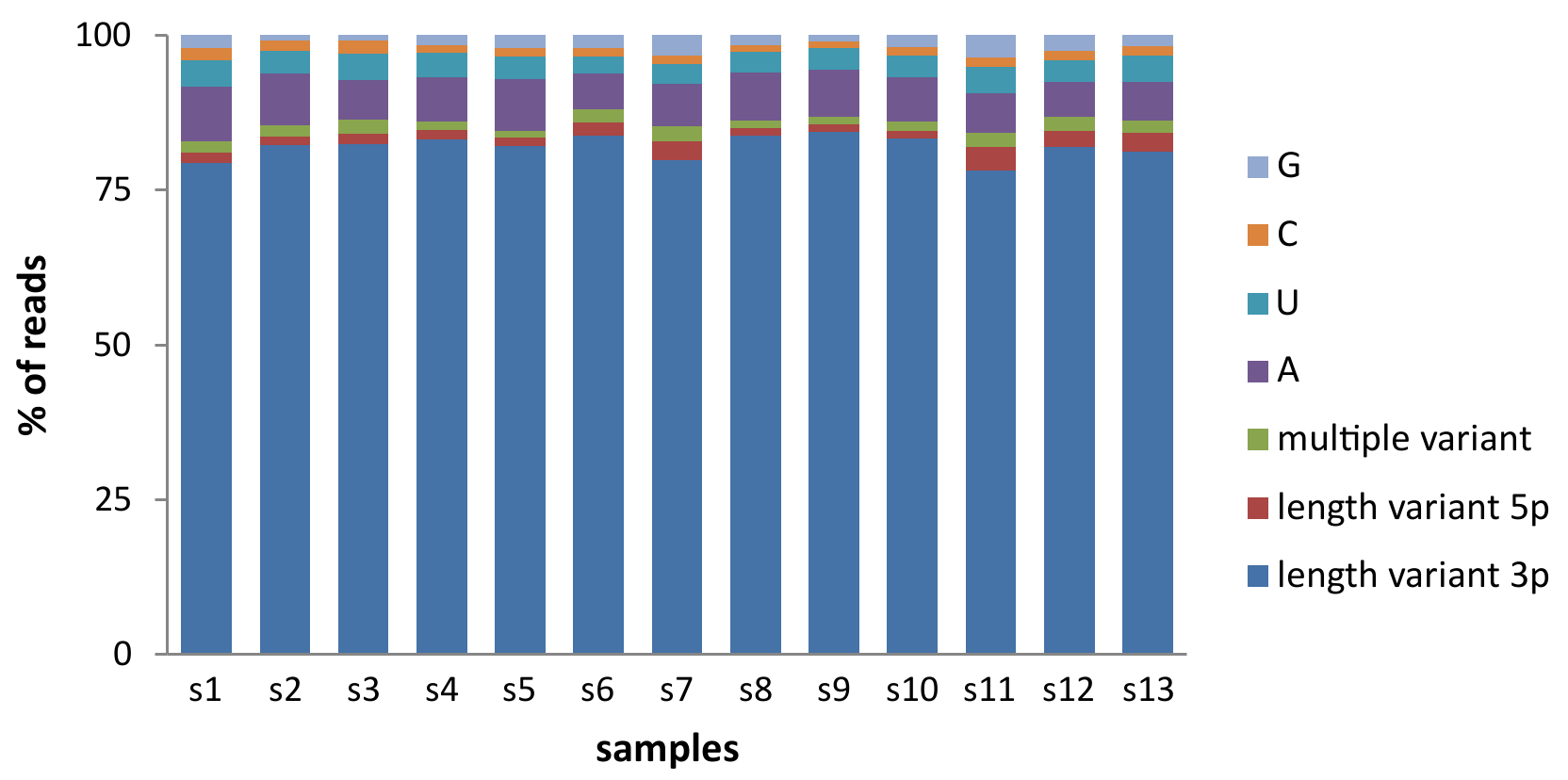
**

**S3 Fig. Multiple sequence variants and non-templated additions of all isomiRs detected.** Proportions of 3’,5’, multiple sequence variants and non-templated additions (G, C, T, A) of all isomiRs detected in samples s1 through s13.
