# Supplementary material for: Illumina Sequencing Reveals Aberrant Expression of MicroRNAs and Their Variants in Whitefish (Coregonus lavaretus) Liver after Exposure to Microcystin-LR
Source: PLoS One. 2016 Jul 8;11(7):e0158899. doi: 10.1371/journal.pone.0158899 (PMC4938405; doi:10.1371/journal.pone.0158899)
Supplement: S1 File — (DOCX) [file pone.0158899.s004.docx]

**S1 File. Confirmatory qPCR study.**

With regards to the microRNAs (miRNAs) expression analysis, we designed a protocol based on polyadenylated RNA and stem-lopp reverse transcription (Biggar et al. 2014) followed by Real-Time PCR. The principle of this procedure is to add an additional polyadenylate tail to the mature miRNAs. Extended miRNAs are reverse transcribed into cDNA using a universal long stem-loop primer that contains a repeat of thymine nucleotides. In the next step, cDNA is amplified with miRNA-specific forward and universal reverse primers in Real-Time PCR. In this study, miRNA-specific forward primers were designed mostly based on the conserved 5' miRNA ends of *Salmo Salar* database (Table 1).

*Total RNA extraction and reverse transcription (RT)*

Total RNA was isolated from the livers of control and MC-LR-treated whitefish using a mirVana isolation kit (Life Technologies; USA). miRNA polyadenylation was performed using a polymerase tailing kit (Epicentre). Reactions were prepared with 1 μL of 10x polyadenylate polymerase buffer, 1 μL of adenosine triphospate (ATP, 10 mM), 0.5 μL of *Escherichia coli* poly(A)polymerase (4U), 1 μg of total RNA, and RNase-free water to a final volume of 10 μL. Reaction mixtures were incubated at 37 °C for 30 min, followed by 95 °C for 5 min to terminate the adenylation, and then transferred directly to ice. Reverse transcription was performed using a RevertAid^TM^ First Strand cDNA Synthesis Kit (Thermo Scientific). An aliquot of 10 μL of polyadenylated RNA from the previous step was incubated with 1 μL of 100 μM universal stem-loop RT primer (5`-CTC ACA GTA CGT TGG TAT CCT TGT GAT GTT CGA TGC CAT ATT GTA CTG TGA GTT TTT TTT TVN-3`). The reaction mixture was heated at 95 °C for 5 min to denature the RNA, and then incubated for 5 min at 60 °C to anneal the stem-loop primer, and finally, it was cooled on ice for 1 min. After cooling, the remaining reagents were added: 4 μL of 5x reaction buffer, 20 U of RiboLock RNase Inhibitor, 2 μL of dNTP mix, and 200 U of RevertAid M-MuLV Reverse Transcriptase. The reaction proceeded for 5 min at 25 °C, followed by 60 min at 42 °C and 70 °C for 5 min. RT product was diluted (100x) and stored at -80 °C.

*qPCR*

Real-Time PCR was performed on an ABI 7500 Real-Time PCR System (Applied Biosystems). For quantification of miRNAs, each 10 μL of Real-Time PCR reaction mixture consisted of 5 μL of Power SYBR® Green PCR Master Mix (Applied Biosystems), optimized concentration of forward and reverse primer (Table 1) and 0.5 μL of the diluted cDNA as a template. Reactions were incubated in 96-well plates at 95 °C for 10 min, followed by 40 cycles of 95 °C for 15 s and 60 °C for 1 min. On the plate, NTC (no template control) and RT-negative samples were included to rule out the possibility of cross-contamination. Melting curve analysis followed by agarose gel electrophoresis was performed to verify the quality of the obtained PCR products. Quantitative cycle (Cq) values were generated on the 7500 System software (SDS 1.3; Applied Biosystems).

Table 1. Details of the oligonucleotides used in this study.

| Name | miRbase acc. No. | Sequence (5’→3’) | Final concentration (μM) |
| --- | --- | --- | --- |
| MiR10b-5p | MIMAT0032295 | acactccagctgggtaccctgtagaaccga | F: 1 |
| Universal Reverse | - | ctcacagtacgttggtatccttgtg | R: 0.25 |
| MiR21a-3p | MIMAT0032534 | acactccagctgggcgacaacggtctgtaa | F: 1 |
| Universal Reverse | - | ctcacagtacgttggtatccttgtg | R: 0.25 |
| MiR23a-3p | MIMAT0032549 | acactccagctgggatcacattgccaggga | F: 1 |
| Universal Reverse | - | ctcacagtacgttggtatccttgtg | R: 0.25 |
| MiR92b-3p | MIMAT0032712 | acactccagctgggtattgcactcgtccc | F: 0.5 |
| Universal Reverse | - | ctcacagtacgttggtatccttgtg | R: 0.5 |
| MiR122-5p | MIMAT0032301 | agctgggtggagtgtgacaatggtgtttg | F: 0.5 |
| Universal Reverse | - | ctcacagtacgttggtatccttgtg | R: 0.5 |
| MiR122-2-3p | MIMAT0032303 | acactccagctgggaacgccattatcacac | F: 1 |
| Universal Reverse | - | ctcacagtacgttggtatccttgtg | R: 0.25 |
| MiR152-5p | MIMAT0032385 | acactccagctgggcaagttctgtgataca | F: 1 |
| Universal Reverse | - | ctcacagtacgttggtatccttgtg | R: 0.25 |
| MiR221-3p | MIMAT0032538 | acactccagctgggacctagcatacaatgt | F: 0.5 |
| Universal Reverse | - | ctcacagtacgttggtatccttgtg | R: 0.5 |
| MiR181a-5p | MIMAT0032417 | acactccagctgggaacattcaacgctgtc | F: 1 |
| Universal Reverse | - | ctcacagtacgttggtatccttgtg | R: 0.25 |
| MiRlet7b-5p | MIMAT0032701 | acactccagctgggtgaggtagtaggttgt | F: 1 |
| Universal Reverse | - | ctcacagtacgttggtatccttgtg | R: 0.25 |

*Calculations and statistical analysis*

Real-Time PCR efficiency was estimated by running reactions with the designed primer pairs (Table 1) and a series of cDNA dilutions as template. Then, Cq values obtained in this way were plotted against cDNA concentration to calculate the slope, and the corresponding efficiencies were calculated according to the equation: E = 10^[− 1/slope]^ (Pfaffl, 2001).

The Cq values were analyzed using GenEx 5 Professional software (MultiD Analyses; Sweden). The data from the Control and Sham-control groups were pooled together and served as a control group in further calculations or statistical analyses. First, we analyzed the stability of the miRNA expression within and between the experimental groups using NormFinder algorithm (GenEx 5 Professional). Based on variance of the Cq values from the control and the MC-LR-treated samples after 14 and 28 days of the experiment, the software indicated MiR181a-5p and MiRlet7b-5p as an optimal pair of reference miRNAs (Table 2).

Table 2. Stability of the miRNA expression within (Intragroup variance) and between (Intergroup variance) the two experimental groups assessed using NormFinder (GenEx 5 Professional software; MultiD Analyes). Optimal pair of reference miRNAs were highlighted green.

| Name | 14 days | | | |  | 28 days | | | |
| --- | --- | --- | --- | --- | --- | --- | --- | --- | --- |
|  | Intragroup | | Intergroup | |  | Intragroup | | Intergroup | |
|  | Control | MC-LR | Control | MC-LR |  | Control | MC-LR | Control | MC-LR |
| MiR221-3p | 0.2036 | 0.0369 | 0.3558 | -0.3558 |  | 0.3018 | 0.7744 | 0.6504 | -0.6504 |
| MiR92b-3p | 0.0686 | 0.3493 | -0.9379 | 0.9379 |  | 0.0377 | 0.2715 | -1.0471 | 1.0471 |
| MiR152-5p | 0.3967 | 0.0983 | -0.6404 | 0.6404 |  | 1.1168 | 0.5082 | -0.3749 | 0.3749 |
| MiR10b-5p | 0.2257 | 0.3885 | 1.0330 | -1.0330 |  | 0.4400 | 0.2436 | 0.6479 | -0.6479 |
| MiR122-5p | 0.1521 | 1.3067 | -1.8729 | 1.8729 |  | 0.1913 | 0.2237 | -0.8493 | 0.8493 |
| MiR21a-3p | 0.1273 | 0.6676 | 1.1780 | -1.1780 |  | 0.0753 | 0.2935 | 0.5979 | -0.5979 |
| MiR23a-3p | 0.1374 | 0.2081 | 1.1855 | -1.1855 |  | 0.4091 | 0.2702 | 1.0154 | -1.0154 |
| MiR122-2-3p | 0.1224 | 1.4867 | -1.4376 | 1.4376 |  | 0.2730 | 0.7891 | -0.5628 | 0.5628 |
| MiR125b-1-3p | 0.3347 | 0.2872 | 1.0264 | -1.0264 |  | 0.2943 | 0.4499 | -0.0287 | 0.0287 |
| MiR181a-5p | 0.0368 | 0.1096 | -0.0464 | 0.0464 |  | 0.1039 | 0.0669 | -0.1746 | 0.1746 |
| MiRlet7b-5p | 0.0604 | 0.0392 | 0.0567 | -0.0567 |  | 0.2197 | 0.2665 | -0.1490 | 0.1490 |

Then, Cq values obtained for all the samples were normalized against the two selected reference genes. The normalized values were used to calculate the expression of 10 target miRNAs, presented as the fold change relative to the control group (GenEx 5 Professional).

Finally, the data were analyzed for differences between the experimental groups (control vs. treated) using two-tailed *t*-test (independent pairwise comparisons in each exposure period) and considered to be significant at p < 0.05. In the case of groups with unequal variances (Levene's test; p < 0.05), p-values obtained by the *t*-test were corrected by not using pooled estimate for the error term for the *t*-statistic and making adjustments to the degrees of freedom using the Welch-Satterthwaite method. In addition to the *t*-test, Mann-Whitney *U* test was used for pairwise comparisons of groups with non-normally distributed data (Kolmogorov-Smirnov test; p < 0.05). The statistical tests were performed using SPSS Statistics v.23 (IBM; USA).

*Validation of the qPCR method*

Table 3 presents results of the qPCR validation study. Real-time PCR efficiencies for the designed primer set was between 86 and 104%, and the reaction specificity was confirmed by the dissociation analysis and gel electrophoresis that resulted in a sharp peak and a band at the expected length. Among the performed runs, no appreciable target detection (Cq < 40) occurred with the NTC or RT- controls.

References

Biggar KK, Wu CW, Storey KB. High-throughput amplification of mature microRNAs in uncharacterized animal models using polyadenylated RNA and stem-loop reverse transcription polymerase chain reaction. Analytical Biochemistry. 2014;462, 32-34.

Pfaffl MW. A new mathematical model for relative quantification in real-time RT-PCR. Nucleic Acids Res. 2001;29, 2002-2007.

Table 3. Real-time PCR efficiency, and specificity confirmed by dissociation analysis and gel electrophoresis for the designed qPCR primers set.

| Name | MiR10b-5p | MiR21a-3p | MiR23a-3p | MiR92b-3p |
| --- | --- | --- | --- | --- |
| Specificity | 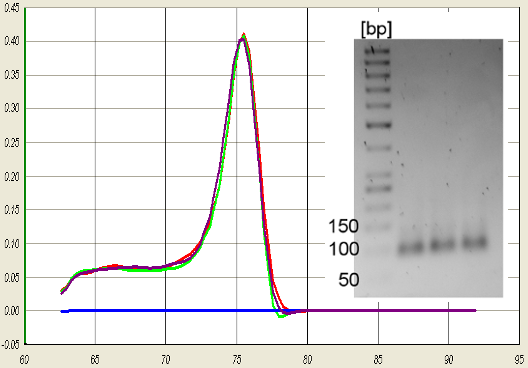 | 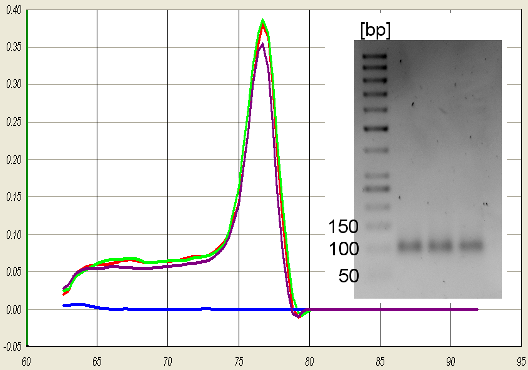 | 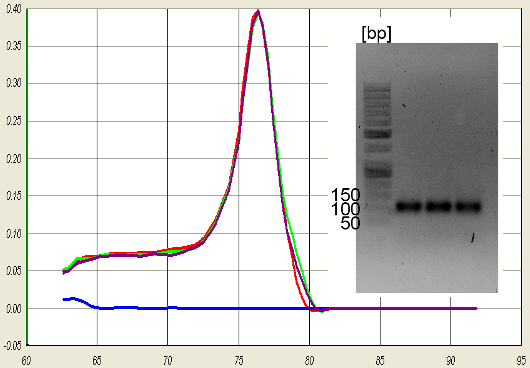 | 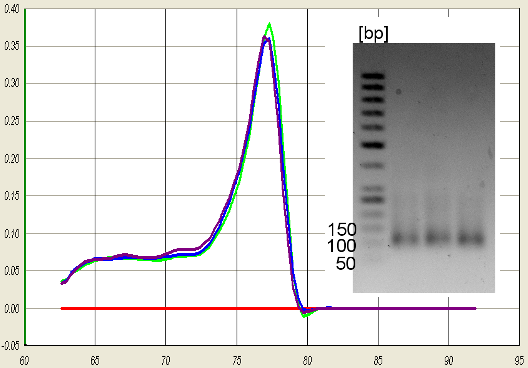 |
| Efficiency | 0.95 | 1.04 | 0.99 | 0.86 |
| Name | MiR122-5p | MiR122-2-3p | MiR125b-1-3p | MiR152-5p |
| Specificity | 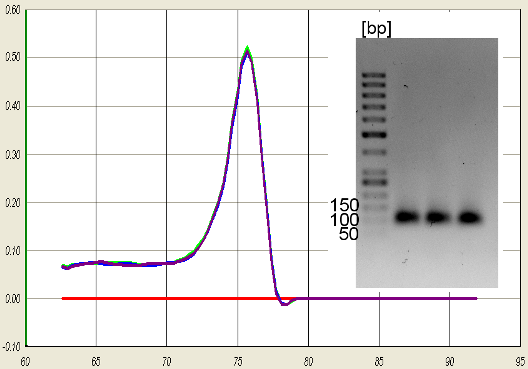 | 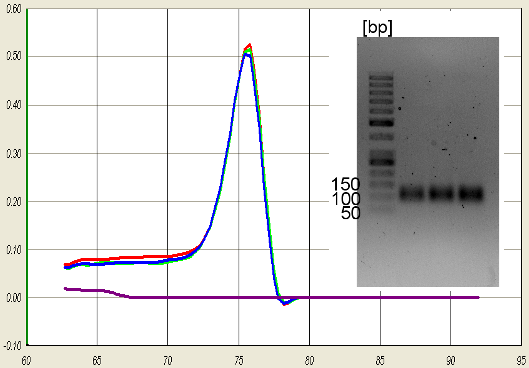 | 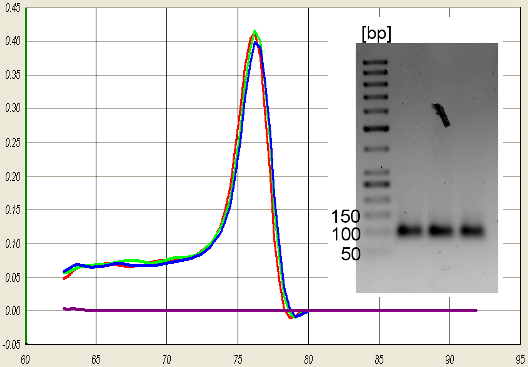 | 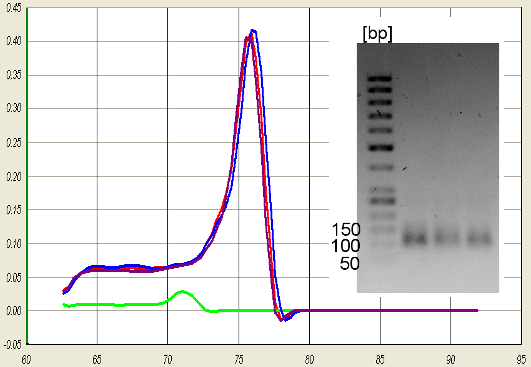 |
| Efficiency | 0.96 | 1.01 | 0.91 | 0.94 |
| Name | MiR221-3p | MiR181a-5p | MiRlet7b-5p |  |
| Specificity | 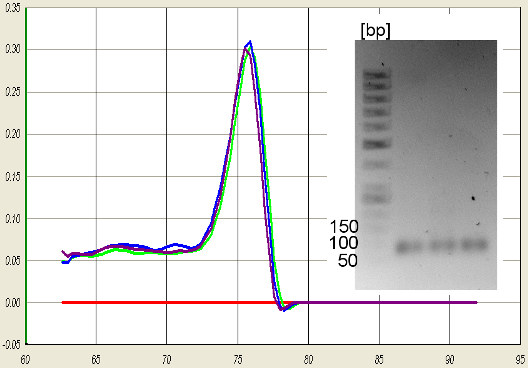 | 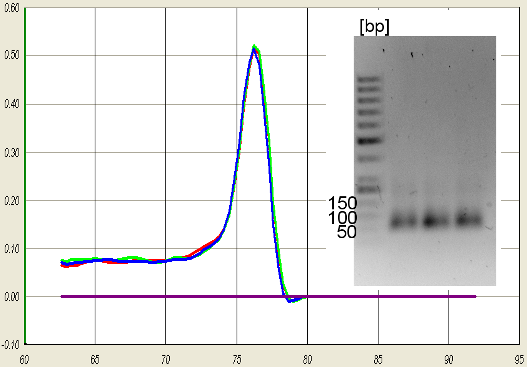 | 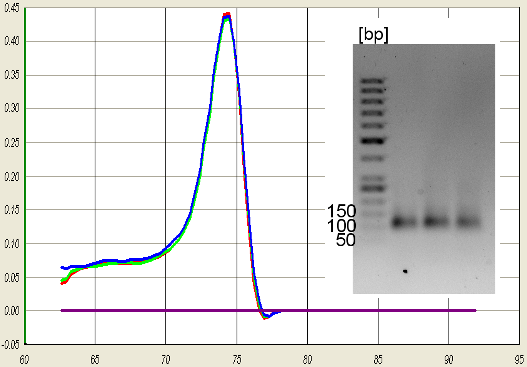 |  |
| Efficiency | 0.91 | 0.92 | 0.90 |  |
