# Supplementary material for: Illumina Sequencing Reveals Aberrant Expression of MicroRNAs and Their Variants in Whitefish (Coregonus lavaretus) Liver after Exposure to Microcystin-LR
Source: PLoS One. 2016 Jul 8;11(7):e0158899. doi: 10.1371/journal.pone.0158899 (PMC4938405; doi:10.1371/journal.pone.0158899)
Supplement: S1 Table — (DOCX) [file pone.0158899.s005.docx]

| Sample Id. | SRS No. | Day/treatment | RNA Concentration ng/µl | RIN | Total trimmed reads | Input reads  (unique reads) | Reads in analysis | Genome mapped reads | miRBase assigned reads  (unique reads) | miRNA (exact and isomiR)  reads |
| --- | --- | --- | --- | --- | --- | --- | --- | --- | --- | --- |
| s_1 | SRR3037124 | 0/no | 338 | 9.8 | 9 550 559 | 5 180 296  (313 404) | 5 177 631 | 4 140 669 | 2 996 281  (16 695) | 2 931 788 |
| s_2 | SRR3043623 | 0/no | 658 | 8.5 | 11 145 359 | 2 652 688  (148 105) | 2 651 340 | 1 771 003 | 1 093 026  (10 193) | 1 087 749 |
| s_3 | SRR3081459 | 0/no | 445 | 9.0 | 9 891 779 | 2 982 862  (139 630) | 2 981 314 | 2 445 499 | 1 760 084  (12 993) | 1 759 504 |
| s_4 | SRR3081466 | 14D/no | 300 | 9.7 | 10 907 572 | 6 476 687  (523 015) | 6 473 384 | 1 565 520 | 191 978  (4 646) | 188 949 |
| s_5 | SRR3081469 | 14D/no | 980 | 9.7 | 5 243 254 | 1 845 837  (167 188) | 1 844 829 | 1 288 937 | 661 625  (7 691) | 643 216 |
| s_6 | SRR3081470 | 14D/yes | 3425 | 9.3 | 10 355 382 | 2 531 977  (168 136) | 2 530 679 | 1 461 909 | 858 081  (9 023) | 849 368 |
| s_7 | SRR3081472 | 14D/yes | 2325 | 9.2 | 7 220 477 | 2 155 635  (128 876) | 2 154 487 | 1 334 139 | 815 433  (8 596) | 808 995 |
| s_8 | SRR3081514 | 28D/no | 530 | 9.2 | 5 703 034 | 3 542 007  (145 148) | 3 540 127 | 2 726 719 | 1 175 940  (9 960) | 1 147 788 |
| s_9 | SRR3081523 | 28D/no | 675 | 8.1 | 11 180 539 | 3 809 195  (446 092) | 3 807 256 | 2 671 859 | 492 456  (6 574) | 482 606 |
| s_10 | SRR3081544 | 28D/no | 602 | 9.1 | 5 298 797 | 1 592 144  (91 468) | 1 591 297 | 1 251 521 | 729 145  (8 183) | 718 456 |
| s_11 | SRR3085234 | 28D/yes | 1200 | 9.8 | 8 001 260 | 2 188 795  (152 710) | 2 187 580 | 1 659 468 | 667 484  (8 258) | 657 175 |
| s_12 | SRR3085243 | 28D/yes | 1080 | 8.8 | 6 134 297 | 1 111 243  (111 491) | 1 110 687 | 743 297 | 232 977  (5 149) | 229 836 |
| s_13 | SRR3085245 | 28D/yes | 880 | 9.9 | 9 650 328 | 2 785 536  (173 531) | 2 784 127 | 1 947 316 | 627299  (7 908) | 606 787 |
| Total  % of input reads No. | | | | | 110 282 637 | 38 854 902  100.00  (100.00) | 38 834 738  99.95  ---- | 25 007 856  64.36  ---- | 12 302 565  31.66  (4.28) | 12 112 217  31.17  ---- |

**S1 Table. Summary of samples sequenced for discovery of *Coregonus lavaretus* miRNAs.**
